# Towards a Post-Graduate Level Curriculum for Biodiversity Informatics. Perspectives from the Global Biodiversity Information Facility (GBIF) Community

**DOI:** 10.3897/BDJ.9.e68010

**Published:** 2021-10-07

**Authors:** Fatima Parker-Allie, Francisco Pando, Anders Telenius, Jean C. Ganglo, Danny Vélez, Mark John Gibbons, Alberto Talavan, Melianie Raymond, Laura Russell, Gautam Talukdar, Manuel Vargas, Raoufou Radji, Hanna Koivula, André Heughebaert, Dag Endresen, Daniel Amariles-García, Takeshi Osawa

**Affiliations:** 1 South African National Biodiversity Institute, Cape Town, South Africa South African National Biodiversity Institute Cape Town South Africa; 2 Real Jardin Botanico -CSIC, Madrid, Spain Real Jardin Botanico -CSIC Madrid Spain; 3 Swedish Museum of Natural History, Stockholm, Sweden Swedish Museum of Natural History Stockholm Sweden; 4 Laboratoire des Sciences Forestières, Faculté des Sciences Agronomiques, Université d'Abomey-Calavi, Abomey-Calavi, Benin Laboratoire des Sciences Forestières, Faculté des Sciences Agronomiques, Université d'Abomey-Calavi Abomey-Calavi Benin; 5 Instituto Alexander von Humboldt, Bogotá, Colombia Instituto Alexander von Humboldt Bogotá Colombia; 6 University of Western Cape, Cape Town, South Africa University of Western Cape Cape Town South Africa; 7 Independent Consultant, Copenhagen, Denmark Independent Consultant Copenhagen Denmark; 8 GBIF, Copenhagen, Denmark GBIF Copenhagen Denmark; 9 University of Kansas, KU Biodiversity Institute, Lawrence, United States of America University of Kansas, KU Biodiversity Institute Lawrence United States of America; 10 VertNet, Lawrence, United States of America VertNet Lawrence United States of America; 11 Wildlife Institute of India, Dehradun, India Wildlife Institute of India Dehradun India; 12 Instituto Nacional de Biodiversidad, Santo Domingo de Heredia, Costa Rica Instituto Nacional de Biodiversidad Santo Domingo de Heredia Costa Rica; 13 University of Lome, Lome, Togo University of Lome Lome Togo; 14 CSC- IT Centre for Science, Espoo, Finland CSC- IT Centre for Science Espoo Finland; 15 Belgian Biodiversity Platform, Bruxelles, Belgium Belgian Biodiversity Platform Bruxelles Belgium; 16 University of Oslo, Oslo, Norway University of Oslo Oslo Norway; 17 GBIF Norway, Oslo, Norway GBIF Norway Oslo Norway; 18 International Center for Tropical Agriculture, Cali, Colombia International Center for Tropical Agriculture Cali Colombia; 19 Tokyo Metropolitan University, Tokyo, Japan Tokyo Metropolitan University Tokyo Japan

**Keywords:** biodiversity informatics, curriculum, policy, data analytics, data management, research agenda, data use, data mobilisation

## Abstract

Biodiversity informatics is a new and evolving field, requiring efforts to develop capacity and a curriculum for this field of science. The main objective was to summarise the level of activity and the efforts towards developing biodiversity informatics curricula, for work-based training and/or academic teaching at universities, taking place within the Global Biodiversity Information Facility (GBIF) countries and its associated network. A survey approach was used to identify existing capacities and resources within the network. Most of GBIF Nodes survey respondents (80%) are engaged in onsite training activities, with a focus on work-based professionals, mostly researchers, policy-makers and students. Training topics include data mobilisation, digitisation, management, publishing, analysis and use, to enable the accessibility of analogue and digital biological data that currently reside as scattered datasets. An initial assessment of academic teaching activities highlighted that countries in most regions, to varying degrees, were already engaged in the conceptualisation, development and/or implementation of formal academic programmes in biodiversity informatics, including programmes in Benin, Colombia, Costa Rica, Finland, France, India, Norway, South Africa, Sweden, Taiwan and Togo. Digital e-learning platforms were an important tool to help build capacity in many countries. In terms of the potential in the Nodes network, 60% expressed willingness to be recruited or commissioned for capacity enhancement purposes. Contributions and activities of various country nodes across the network have been highlighted and a working curriculum framework has been defined.

## Introduction

Biodiversity Informatics (BDI) is a new and developing field of science, with the earliest citation dating back to only 1998 (Schalk 1998). BDI deals with the interrelated challenges of collection, collation, curation, integration, analysis, prediction and dissemination of data and information related to the diversity of life on Earth so as to provide information for decisions on biodiversity conservation and sustainable use (Hardisty et al. 2013, Hobern et al. 2012, Walters and Scholes 2016). Over the last few decades, development of key software tools, such as MaNis (Stein and Wieczorek 2004), Lifewatch (Fuentes and Fiore 2014) and the Integrated Publishing Toolkit (Robertson et al. 2014) for managing, cleaning and publishing data (Hill et al. 2010, Endresen and Knüpffer 2012, Wieczorek et al. 2012), as well as the establishment and functioning of the Global Biodiversity Information Facility (GBIF), were major steps towards sharing biodiversity data globally via the Internet.

Biodiversity Informatics-based knowledge management systems and tools, like the GBIF data portal, the Atlas of Living Australia, Ocean Biodiversity Information System (OBIS), Lifewatch etc., enable users to navigate and put to use vast quantities of biodiversity information, thereby advancing scientific research in areas, such as agriculture, biomedicine, biotechnology, environmental management, pest control, health, education and conservation (GBIF Secretariat 2019), amongst others; serving the economic and quality-of-life interests of society; and providing a basis from which our knowledge of the natural world can grow rapidly and in a manner that avoids duplication of effort and expenditure (OECD 1999). With many critical areas of applied science linked to human well-being like climate change, invasive alien species, agrobiodiversity, health and food security, affected by such a new and dynamically evolving field, the need for increased capacity enhancement in this area of science is important.

This recent and multidisciplinary field of science poses enormous challenges in training, recruitment and retention of personnel. An increase in the amount of skilled personnel to manage biodiversity data and the growing digital assets of organisations is also needed. Furthermore, graduates entering the working environment often do not have the combination of skills required to support the current practice in the workplace, with further work-based training then being required. This need for training may, however, be compromised by organisations which offer staff limited or no professional development opportunities.

Biodiversity informatics also remains largely without any formal synthesis, guide, textbook or summary. That is, no textbook dedicated to Biodiversity Informatics as a science exists to guide users through the steps involved and techniques used. Further, almost no undergraduate or postgraduate programmes exist that provide a comprehensive curriculum for the field (Peterson and Ingenloff 2015), except for a few emerging efforts. At the University of Abomey-Calavi, in Benin, a full Masters level degree in biodiversity informatics, has been initiated in October 2017 (Ganglo 2017a, Ganglo 2017b). The Biodiversity Informatics Training Curriculum (BITC) was initiated in 2012 by the University of Kansas, as a series of detailed training courses which were held across numerous sites in Africa (Peterson and Ingenloff 2015). Video recordings, as well as presentations and lecture materials, of these courses were produced and uploaded on to Youtube and the full set of courses were presented as video and pdf formats teaching curriculum for the field (Peterson and Ingenloff 2015). A second phase of this effort is now underway, led by the University of Oxford (University of Oxford 2018, Ingenloff et al. 2019). In South Africa, at the University of Western Cape (UWC), a 6-8 week Bachelor of Science Honours Elective with a focus on Biodiversity Information Management, has been available since 2012 as part of the Biodiversity and Conservation Biology (BCB) Honours degree. In addition, at UWC, biodiversity informatics components are included in the overall undergraduate degree, as part of the Environment and Sustainability Studies (ESS) course, which is a cross faculty degree, managed by the BCB Department. There are also some emerging efforts at the University of Arizona with a Biodiversity Informatics graduate short course in place, aimed at biology, engineering and information science students at the School of Information (University of Arizona 2021). At the University of Florida, the Integrated Digitized Biocollections (iDigBio) and National Science Foundation (NSF) funded Biodiversity Literacy in Undergraduate Education (BLUE) are initiatives focused on providing training courses and the development of curriculum to support biodiversity data literacy and to grow a network of biodiversity researchers, data scientists and data-centric biodiversity educators (Ellwood et al. 2020).

The current and traditional academic status of biodiversity informatics is that Information and Communications Technology (ICT) graduate programmes equip students with computational and informatics skills, whereas a traditional science degree equips graduates with the basics of biodiversity science. It is the combination of skills derived from the fields of both informatics and biodiversity that is at the core of the skill-set required by the biodiversity information scientist (Martellos et al. 2012). This, especially as new technologies (e.g. mobile-cellular applications, the “internet of things”, citizen scientist data) have created an exponential increase in the volume and types of data available, has created unprecedented possibilities for society and for conservation efforts (IEAG 2014, Osawa 2019). Biodiversity informatics tools and techniques become an enabler for the data-science-policy value chain, from monitoring biodiversity data and priorities, developing species and ecosystem assessments, developing biodiversity scenarios through analysis and modelling and effecting change through impacting governmental policy and supporting sustainable development goals (SDGs).

This paper proposes a framework for a globally-relevant biodiversity informatics curriculum, with contributions from the GBIF biodiversity information management community. It summarises the outcomes of a survey which was conducted prior to the GBIF Governing Board Nodes meeting held in Madagascar in October 2015 and provides recommendations for taking forward efforts in this area of work. The purpose of this paper is, therefore, to further disseminate the survey results and to advance efforts towards developing biodiversity informatics curricula content and to grow biodiversity informatics as a field of science. The initial survey results are summarised, some recommendations are outlined and the preliminary working curriculum framework is defined.

**Policy Context**

The availability of biodiversity data and information and our ability to use this information effectively, demonstrates a value for the evolving field of biodiversity informatics. Developing capacity in efficient mobilisation, management, publishing and use supports national strategies, which feed into international initiatives, including the Convention on International Trade in Endangered Species (CITES), Intergovernmental Platform on Biodiversity and Ecosystem Services (IPBES), Convention on Biological Diversity (CBD) Aichi Targets, United Nations Convention to Combat Desertification (UNCCD), United Nations Framework Convention on Climate Change (UNFCCC) and the Sustainable Development Goals (SDGs), which all require relevant, reliable and accurate data.

GBIF plays a unique role amongst international organisations in biodiversity informatics. It is the only body supported by national governments with a mandate for mobilisation and management of primary biodiversity data on all taxonomic groups, through the GBIF Memorandum of Understandings (MoUs) signed by government entities (GBIF 2011). At the national level, GBIF Nodes are the key coordinating bodies for GBIF-related biodiversity informatics activities (data mobilisation, management, use and publishing) in participant countries (GBIF Secretariat 2020). Capacity enhancement has, therefore, been identified as critical to enabling the sharing of fit-for-use data, to fill data and knowledge gaps.

In March 2018, GBIF and IPBES signed a MoU, to ensure collaboration and that the activities of the two networks are complementary and closely aligned*^1^. IPBES is an intergovernmental body which assesses the state of biodiversity and of the ecosystem services it provides to society. It thus produces data (although not raw primary data) and knowledge products to support the science-policy interface. Improving the availability of data and scientific information on biodiversity will strengthen the ability of countries to contribute to and benefit from IPBES activities, including the regional and thematic assessments*^2^. A number of strategic areas for collaboration relating to the data landscape can, therefore, be identified, with GBIF as a knowledge management platform and a resource for primary biodiversity data, including: 1) helping IPBES to identify and access biodiversity datasets relevant to IPBES assessments and indicators; 2) using knowledge gaps identified through IPBES assessments to help prioritise mobilisation of new data through GBIF’s network of nodes and data publishers; 3) encouraging collaboration between GBIF’s national nodes and IPBES national focal points; and 4) coordinating GBIF’s capacity building activities to support data mobilisation and access relevant to IPBES.

Globally, an overwhelming majority of countries are signatory to the CBD and have strived to achieve the twenty Aichi Targets for biodiversity conservation, through the Global Strategic Plan for Biodiversity 2011-2020 (CBD 2010). GBIF is an observer to the CBD and actively participates on several Technical Committees. As a member of the Biodiversity Indicators Partnership (BIP), the number of species occurrence records published through GBIF provides an indicator of progress towards Aichi Target 19 on sharing of data and knowledge*^3^. This may be disaggregated to national or regional level.

Building capacity in biodiversity informatics would, therefore, respond directly to globally agreed priorities for accelerated action to achieve targets on biodiversity conservation and sustainable development. This latter ultimately aims to end poverty, increase food security, promote education, fight inequality and injustice and tackle climate change. Capacity enhancement through either work-based or academic training is, therefore, critical.

## Material and methods

A GBIF Nodes thematic group was established prior to the GBIF Nodes Meeting in October 2015, with the aim of getting a clear idea of how the GBIF biodiversity informatics community is contributing to development and implementation of biodiversity informatics curricula. Such curricula would support both work-based training of biodiversity informatics professionals and academic teaching at academic institutions, in their national, regional or global contexts. Specifically, it was intended to get an idea of how the GBIF community can contribute to the growing field of biodiversity informatics going forward.

Two interventions were planned and executed before the 2015 GBIF Nodes meeting, to assess current levels of activity in the GBIF network. Interventions included: (1) A survey conducted before the meeting and (2) two curricula frameworks disseminated for comment and input.

**Survey Method**

The survey was divided into two components, a first part with six questions that all node managers were invited to answer and a second part with ten more detailed questions to be answered by those with a higher activity level in the field. The survey, including the responses by participants, is provided as supplementary information.

**Frameworks: Curricula for Biodiversity Informatics**

Two curriculum frameworks were provided as a basis for discussion, each aiming to outline comprehensive BDI courses at the Honours or Masters level or for professional work-based training. The two documents included the Biodiversity Informatics Training Curriculum Version 1.2 (Peterson and Ingenloff 2015) and a parallel curriculum prepared by Parker-Allie as a component of a research study, to produce a conceptual framework for developing the field of biodiversity informatics in South Africa. The production of the second curriculum (Parker-Allie et al., in prep), is part of an initiative to develop a curriculum for further implementation, as part of a 5-year strategy to develop capacity in biodiversity informatics in South Africa*^4^.

In the facilitated session at the GBIF Nodes meeting, existing initiatives across the broader community were also considered, highlighted and discussed, including activity by the Biodiversity Information Standards TDWG Interest Group on Biodiversity Informatics Curriculum*^5^ and the Biodiversity Informatics Training Curriculum*^6^.

## Data resources

The results of the survey is presented here as supplementary information in Suppl. material 1.

## Results

**Part One of the Survey**

**Figure 1.**
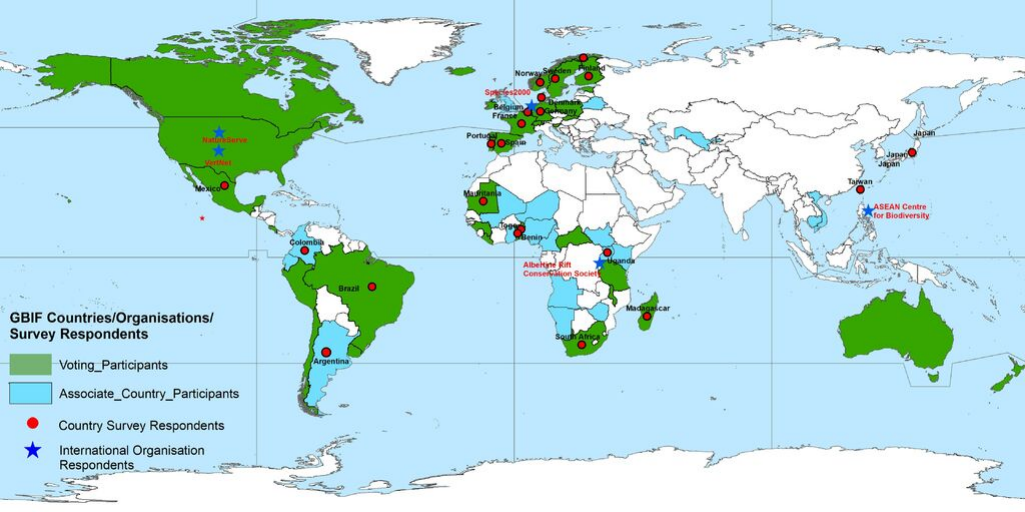
Map depicting GBIF Voting and Associated Countries, as well as the survey respondents from GBIF Nodes and GBIF Associated International Organisations.

Responses were received from 31 individuals in 23 countries (GBIF Nodes) and five international and intergovernmental organisations from five continents (Fig. 1). This respondent pool is a small, but diverse group of respondents representing a wide array of professional positions, including Node Managers and/or Nodes staff. The respondents to the survey also occupy a range of professional positions including professors (all ranks), scientists, database specialists, ICT specialists and knowledge system engineers, directors, managers etc. The survey also indicated that some individuals support GBIF-related activities on a full-time basis, whereas for others, this may only be a part-time activity.

**Figure 2.**
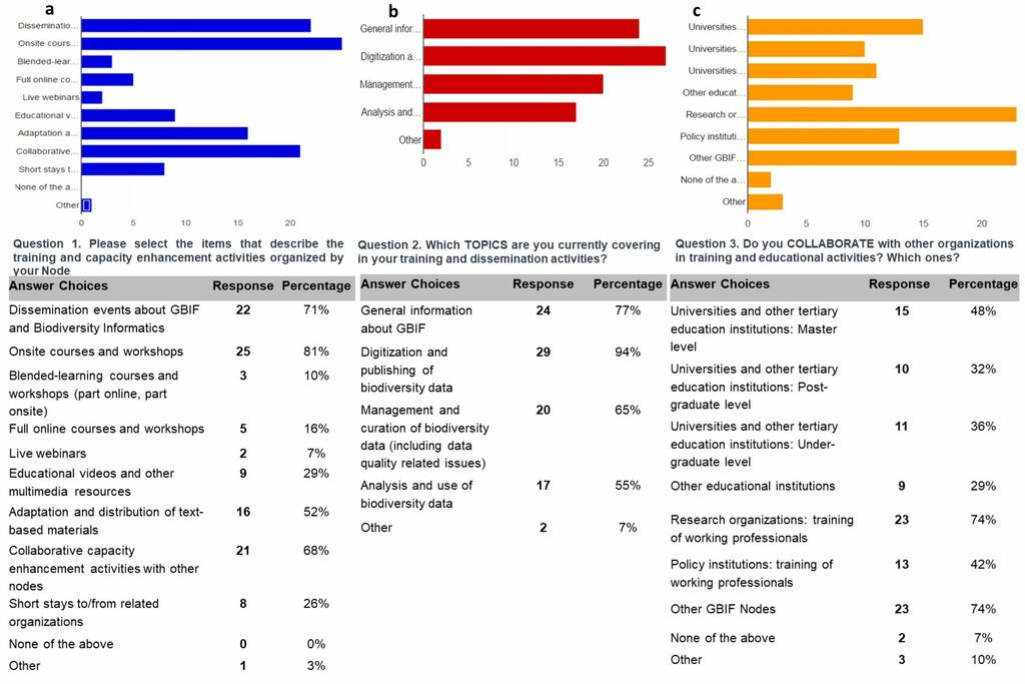
The training and capacity enhancement activities of the GBIF Nodes (questions detailed in the survey are included as supplementary information): a) Q1 describes the training and capacity enhancement activities of the Node; b) Q2 topics currently being covered by training and capacity enhancement activities; c) Q3 shows organisations with which Nodes are collaborating in relation to training and capacity enhancement activities.

The first component of the survey showed the training and capacity enhancement activities of the GBIF Nodes (Fig. 2). Results indicated that 80% of GBIF Nodes conduct on-site courses and workshops related to GBIF and biodiversity informatics (Fig. 2a), often (68%) as collaborative capacity enhancement activities with other Nodes. Survey respondents use various mechanisms to enable training, with > 50% using text-based materials and almost 30% using various digital means, such as online platforms and courses, live webinars, educational videos and multimedia resources. About 16% of respondents indicated that they were conducting full online courses; only 7% indicated that live webinars are being presented.

Most topics covered in Node training activities included principally digitisation and publishing of biodiversity data, followed by management, curation and analysis of biodiversity data (Fig. 2b). Training by the informatics community is predominantly focused on the training of work-based professionals, with research and policy institutions making up approximately 74% and 42%, respectively, of the target audience (Fig. 2c). Other undergraduate and postgraduate students, based at universities and other tertiary education institutions, make up 36-48% of the core audience for training and capacity building efforts.

Survey respondents were asked to provide a reference, including a URL link or publication, to their training and dissemination activities (question 5 of survey). Table 1 provides a list of websites, based on the survey outcomes, that can be accessed and explored for relevant events, initiatives, tools and training resources, for further dissemination of new knowledge related to biodiversity informatics theory and practice. Further to this list, a number of other highly relevant URLs, which were also identified in the facilitated discussion session at the Nodes Meeting in Madagascar in 2015, also provide accessibility to key training resources including https://www.gbif.org/resource/, as well as the https://datacarpentry.org/lessons/ which includes various domains, such as ecology, biology and genomics curricula. The http://biodiversity-informatics-training.org/ is a fundamental resource for biodiversity informatics teaching and training. Here a number or training modules, with available training resources, such as presentations and video recordings, have been developed and are freely available for download. The courses and content provided include topics such as building biodiversity informatics institutions, data capture, data cleaning, data publishing, data analysis, national biodiversity diagnoses, species descriptions, ecological niche modelling and public health applications. Another very useful resource identified outside the survey results, includes the URL of the Integrated Digitized Biocollections (iDigBio), the National Resource for Advancing Digitization of Biodiversity Collections (ADBC) funded by the National Science Foundation https://www.idigbio.org/wiki/index.php/Digitization_Resources.

### Part two of the Survey

**National biodiversity informatics educational activities**

In the second component of this survey, 22 of the 31 initial survey participants continued to the latter half of the survey, which requested more detailed information, at the national level. An initial assessment of academic teaching activities highlighted that several countries, to varying degrees, were already engaged in conceptualisation, development and implementation of formal academic programmes in BDI.

In this regard, both Benin and South Africa engaged in developing postgraduate curricula and implementing teaching activities at the tertiary/university level. Benin has developed a BDI curriculum for an MSc Masters Level course at the University of Abomey-Calavi and initiated implementation in October 2017. South Africa engaged in the rollout of a Biodiversity Information Management course at the BSc Honours level, at the University of Western Cape (2012-2016). In Scandinavian countries, at the time of the survey in 2015, biodiversity informatics was starting to be integrated into university training programmes, including recent academic professorships in biodiversity informatics at the Norwegian University of Science and Technology in Trondheim and the University of Bergen. Survey results also indicated that most Swedish universities are open to teaching or already teach BDI elements to some (basic) extent, but normally not at the undergraduate stage.

Further, in Europe, survey results indicate that the Spanish Node is involved in teaching BDI-related content at the Masters level and in France, the objectives of the Global Biodiversity Information Facility (GBIF) have been introduced into a course on Systematics and Biodiversity Informatics. It is noteworthy that the Scientific Coordinator of GBIF France holds a Professorship at the University of Paris, which can positively impact the field of BDI nationally. It is useful to note also that, while not specifically mentioned as an outcome of the survey, biodiversity informatics curriculum has also been taught in at least one university in Spain, The University of Navarra (UNAV) since approximately 2010, at the undergraduate level and at the Master's level since about 2015.

Survey results have indicated that Latin American countries, such as Brazil and Colombia, have considered BDI courses at the tertiary level. In Colombia, the GBIF Node is working with at least four universities that are willing to adopt courses on this topic. In the facilitated discussion, which took place at the Nodes meeting (which also provided an opportunity for engagement and elaboration of the details of the survey), it was identified that, in Costa Rica, the Instituto Nacional de Biodiversidad (INBIO) is in the process of reinstating a UNESCO Chair in Biodiversity Informatics at the Technological Institute of Costa Rica. Further follow-up discussions with academics also clarified that here, a one-semester graduate-level course called "Introduction to Biodiversity Informatics" is taught annually (E. Mata, personal communication). Followup discussions with academics also identified that, in Mexico, there are three courses at two universities, focused on data analysis. Here, many students do two of the three courses to get up to speed (A. Townsend Peterson, personal communication).

In the Asian region, the survey results indicated that the Wildlife Institute of India has implemented a one-week biodiversity informatics component, within a larger module of landscape ecology in a two-year Masters in Wildlife Science course. The survey also identified educational activities conducted by the Taiwanese and Japan Nodes, through the Biodiversity Information Fund for Asia (BIFA). Follow-up discussions with the Nodes, however, identified that the Taiwanese and Japan Nodes implemented a workshop in biodiversity informatics in 2016 (https://sites.google.com/site/2016bifabic/), which resulted in a draft of a Biodiversity Informatics Cookbook with some teaching slides. The workshop training presentations, as well as the draft Cookbook, are all accessible online*^7^.

Other information reported in the survey indicated that several of the survey participants were also engaged in Masters, PhD and postdoctoral student supervision or management and/or had developed projects to support students.

#### Harnessing the potential of the Nodes Community to support BDI teaching and work-based training

Most survey participants (67%) indicated that they would be willing to make their course content openly available, to support, further rollout and uptake of BDI as a field of science. A few countries are looking at further expanding their efforts in BDI courses and degrees, although constraints here included funding, time and resources. Nodes are involved primarily in work-based training and some have indicated that they could expand their programmes for practitioners, but not necessarily into a more academic (university) context, as academic teaching in BDI was not seen as a core function of GBIF Nodes. However, other European countries, such as Norway, Sweden and France, indicate involvement in student projects and undergraduate training.

On the African continent, countries such as South Africa and Benin have fostered academic partnerships with institutions of higher learning and are facilitating the development of biodiversity informatics as a field of science. In South Africa, an extended curriculum (BSc. Honours) for Biodiversity Information Management has been developed and SANBI is engaging with additional universities to consider the roll-out of a BDI course. SANBI-GBIF has also developed a five-year strategic plan, which includes bringing Honours, Masters and PhD students on board, as well as informatics expertise, at the regional and international level. It has, thus far, been instrumental in funding two postdoctoral students in the area of Biodiversity Informatics Research Strategy development and producing innovative research into relevant biodiversity informatics tools and applications. SANBI-GBIF is also working with national government to put in place a Research Chair for Biodiversity Informatics. In Benin, two Masters level students from the BDI programme upgraded from the 2020 academic year and registered as PhD students in the BDI programme. These students, were amongst the first cohort who successfully achieved their Masters degrees in the programme.

**E-learning**

In part one of the survey, we identified that digital platforms were being used by some countries as a medium to facilitate training. In part 2 of the survey, we identified four Nodes that were using e-learning platforms. Spain has been implementing an e-learning platform since 2010 and 34 online courses have been carried out since then, providing training to 400 students from 16 countries. The platform used by Spain from 2010-2017 was ATutor, which was substituted in 2017 by Chamilo, which allows online access to learning materials and distance-learning courses. In France, content such as data publication, data quality and data papers, are being taught through an e-learning platform and, in other instances, tools such as Adobe Captivate, are being used.

**Research Agenda**

We posed the question to the research community as to what they considered to be the key research questions that can be addressed through biodiversity informatics and what were the global priorities for BDI. Some respondents felt that biodiversity informatics is a methodological science, an approach and methodology that should be used as a tool. One great possibility with informatics is to explore data to find trends that are not visible in existing data. It could, therefore, be used to discover how to make "big data" resources more useful to both scientists and decision-makers, with current and emerging technologies. This could mean development of standards and ontology models (including taxonomies) and methods for annotating data with this information and creating mechanisms for linking this additional value back to the originator of the data.

The research areas identified included very technical components, such as those related to data generation, data standards, publishing and visualisation. Other broad thematic areas identified through the survey, that could be supported through BDI research included: global warming, climate change, invasive alien species, species distribution modelling, taxonomic and phylogenetic analysis, biodiversity conservation strategies, species assessments, such as Red Lists and Black Lists, agrobiodiversity and analysis of species traits.

**Potential contributions and opportunities to a globally offered biodiversity informatics training curriculum**

To assess the level of potential in the network to support academic teaching and work-based training, we enquired to what extent survey respondents were willing to mentor or train professionals. Sixty percent indicated that they would be willing to be recruited or commissioned to support teaching activities, with only 15% indicating they were not available. The expertise of the Nodes network would be very valuable to support ongoing capacity development in BDI broadly, across programmes and country-led initiatives, with the time constraint needing to be considered.

## Discussion

**Outcomes of the facilitated discussion of the GBIF Nodes Meeting**

The facilitated session provided a platform for further discussion and elaboration of the survey results. It also provided an opportunity for further engagement on the existing activities, needs, existing content and resources for training and teaching activities in GBIF Participant countries. Importantly, some clear recommendations and outcomes where identified and are summarised in Table 2.

It was also recommended that a standard modular curriculum, integrating all the existing curricula that already exists, including the results of the survey, be developed. As a consequence, this paper provides a high level working curriculum framework (Table 3), which includes subjects identified as important in a number of biodiversity informatics university courses, including the University of Kansas, University of Abomey-Calavi, University of Mexico, University of Costa Rica, University of Western Cape, Nordic Academy of Biodiversity and Systematic Studies (NABIS).

The working curriculum framework also includes work-based training courses conducted by the GBIF Secretariat and Nodes and spans the data value chain from data generation to fill data and knowledge gaps, to data management and subsequently data use and its applications. The GBIF and the Biodiversity Information Standards (TDWG, from its former name: Taxonomic Databases Working Group) networks can take forward capacity development efforts in BDI. Individuals in these networks are already engaged in developing and improving biodiversity data standards (Wieczorek et al. 2012, Robertson et al. 2014) and in developing principles and workflows in relation to data management and cleaning (Hill et al. 2010). Here, scientists are looking at innovative approaches to the development of new technological platforms and the ongoing improvements and integration of current mega-science initiatives and infrastructures (GBIF 2018). This was highlighted at the 2^nd^ Global Biodiversity Informatics Conference, where the major players agreed to a light coordination mechanism of the major biodiversity informatics platforms (GBIF 2018, Hobern et al. 2019) to improve the availability and quality of biodiversity knowledge in order to observe, measure and model the biodiversity on Earth. Many scientists are also actively engaged in data mining (Ariño 2010, Otegui et al. 2013, Osawa 2019) and analysis of biodiversity data (Asase and Peterson 2016, Ganglo and Kakpo 2016, Freeman and Peterson 2019, Osawa 2019), with much of these efforts synthesised in the annual GBIF Science Review (GBIF Secretariat 2019). It is, therefore, imperative that large scale efforts are made to harness the expertise which exists in BDI and to breach the digital divide between developed and developing countries. Capacity development is one such unifying area that has the power to improve lives through enhanced skills and education.

With regards to data use and applications, this study has demonstrated the need for a research agenda for BDI. Peterson et al. (2010) described broad areas that BDI can support, including: (1) geography and ecology of past life; (2) a biota-wide picture of diversification and interactions; (3) future (novel) communities; (4) integrating phenotype and genotype and (5) synthetic conservation planning (Peterson et al. 2010). The GBIF workplan (2014-2016) also identified some candidate priority areas as potential areas for collaborative delivery by the Nodes, task groups and strategic partners. These included: (1) large‐scale data for biodiversity monitoring and assessment; (2) data to support research and responses relating to invasive alien species; (3) global distribution data for plants; (4) tools to support 21st century taxonomy and (5) global cyber‐infrastructure for all biodiversity information. A number of outputs (methodologies, research papers, best practice reports from task groups) have been identified in relation to some of these research areas (Faulkner et al. 2014), including recommendations from fitness-for-use task groups on agrobiodiversity (Arnaud et al. 2016) and niche modelling (Anderson et al. 2016).

In this fast changing digital environment, new technological advancement is leading to an exponential increase in the volume and types of data available, resulting in a data deluge (The Economist 2010). This presents both an opportunity and a challenge. The accessibility of vast amounts of data, coupled to advances in informatics platforms, brings about the ability to change outcomes (The Economist 2016), to truly empower people all over the world and move some economies towards a more equitable growth path, transforming from what might be a resource-based economy towards a knowledge-based economy (Gonzalez-Brambila et al. 2016). Hardisty et al. 2013 have indicated that the grand challenge for biodiversity informatics is to develop an infrastructure to allow the available data to be brought into a coordinated modelling environment (technological framework of interoperability), able to address questions relating to our use of the natural environment that captures the ‘variety, distinctiveness and complexity' of all life on Earth.

For us to maximise the volumes of data and the technological infrastructure, it is necessary to develop a research agenda and develop a direction for BDI research and development. As research becomes more data driven, it is also our ability to exploit the potential of big data and Information and Communication Technology (ICT) that can shift us towards an economy that is more technologically driven, from the third industrial (digital) revolution into the innovation of the fourth industrial revolution where the physical, digital and biological worlds continue to converge (Department of Science and Technology 2019). This will require us to fully realise the value of Science, Technology and Innovation (STI).

**What does a biodiversity informatics curriculum mean for Higher education**?

Based on the results including the needs expressed by the biodiversity community, it is clear that a standard modular working curriculum is needed (Table 2) which can support both work-based training and academic teaching, to advance the understanding and uptake of biodiversity informatics science, tools, applications and research. There is also a clear need/recommendation for a more formalised approach, in training and teaching of biodiversity informatics globally. Engagement with institutions of higher learning is, therefore, critical to support the development of biodiversity informatics training and teaching. This is further supported by the GBIF strategic plan (GBIF 2017b), which highlights the promotion and inclusion of biodiversity informatics training as part of relevant university and workplace education. Although not explicitly captured in the survey, the inclusion of Library and Information Science Departments and professionals to support the development of the BDI curriculum and teaching will also be beneficial. Information Science Professionals have the underlying data literacy, data management and information science skills needed in the biodiversity informatics community. In addition, they also have systems theory backgrounds that help them understand networks.

To embed a curriculum within a tertiary institution, resources will be required to enable capacity development in BDI. This includes the need for an increase in high level academic experts to grow this field of science, with indications that the interchange of professorships is needed (Table 2). The exchange of high-level expertise is catalytic and presents an opportunity for coursework and curricula to be taught and to establish collaborative research areas, if desired. Travel grants for visiting scientists, offered by funding bodies, would be a worthwhile mechanism for the exchange of expertise i.e. the National Research Foundation (NRF) in South Africa or the National Science Foundation (NSF) of the United States of America, as well as the European DiSSCo initiative which makes funds available for enabling capacity building efforts in BDI. In the case of South Africa and the USA, these are short term grants, however, for stays up to 3 weeks, which is an opportunity, but also a limitation for effective collaboration (National Research Foundation 2019, United States Agency for International Development 2019). However, these types of opportunities should be fully explored by relevant implementation agencies or GBIF Nodes, which has a goal to expand its capacity development focus area and align with universities, to include the development of Biodiversity Informatics Honours, Masters and PhD degrees.

Government funding for visiting scientists may not be an option in many countries. Having a dedicated Research Chair (as a focal point of high-level academic expertise), however, will be a more sustainable mechanism to develop Biodiversity Informatics as a field of science. Through the establishment of research chairs, academic hubs of activity will develop, where the development and implementation of a curriculum for students in conjunction with a more strategic research approach or a research agenda can be achieved. This will drive the understanding of the mobilisation, management, publishing, use, relevance and impact of the data for society and development.

The establishment of a Research Chair in the field of biodiversity informatics, based at a tertiary institution, is likely to enable an environment of innovative and vibrant learning. Such a hub will (ideally) need to accommodate: 1.) A mixture of local, regional and international students to allow for global exchange and interaction; 2.) Facilities to assist students and academics to engage formally and informally (lecture rooms, library, outdoor facilities); 3.) Opportunities to engage in discussion groups regarding biodiversity information management related work or outputs; 4.) Opportunities to engage in seminars and other forums and 5.) Opportunities to attend weekend or week-long field research trips. This allows students to gain valuable field experience and often creates a more informal environment to discuss issues with supervisors or other academics/researchers.

What this would mean for higher education institutions is that curricula will need to be mainstreamed into degree and postgraduate courses, if we want to develop biodiversity informatics as a field of science and if we want to make an impact as a sector. This may take several years to implement and is also often market-driven. In the instance of the Benin Node, developing and implementing a Master’s level curriculum took approximately two years from conceptualisation to implementation. Benin’s advantage was the existence of a Professorship, with the Node being hosted at a tertiary institution like the University of Abomey-Calavi.

Having the academic capacity to provide the teaching required is, therefore, critical, while the need for finding work-based placements for students entering the working sector is equally important and may be a limiting factor for universities in investing in such a course. Availability of data management and biodiversity informatics positions is critical. Ultimately, there needs to be institutional buy-in relating to the need for biodiversity information managers and/or biodiversity information scientists, to ensure the viability in terms of job opportunities. There needs to be some balance in the rate of production of qualified students versus the number of jobs being advertised.

Some further key considerations for biodiversity informatics to become more integrated into Higher Education Sector and be more sustainable, includes the ability to tap into funding in a number of areas including; 1) scholarships to support the socially-disavantaged groups of students; 2) internships and MSc and PhD students; 3) funding to support international teaching missions for academics, work-based professionals and potential trainers and 4) funding to support active research work. The ability to access funding for this range of activity will promote improved efficiency and sustainability with this approach.

**What does a biodiversity informatics curriculum mean for the Science-Policy Interface and Heads of Delegation (HoD) to GBIF**?

Member countries, through their national governments, have committed themselves to supporting GBIF as a mega-science research infrastructure, which has great potential to enhance outputs for science, technology and innovation (STI). Here, we refer to research infrastructures not just as a physical technology structure/platform, but rather as part of a research ecosystem involving whole communities. Thus, in keeping with the original recommendation of the Organisation for Economic Co-operation and Development (OECD), to foster increased cooperation and participation in science, education and enhanced engagement by academia and other communities in biological informatics, the GBIF infrastructure provides a mechanism to support this vision by providing opportunities for STI output and impact, through the use and application of biodiversity data, the development of informatics platforms and the implementation of workflows and methodologies, as well as the implementation of tools and techniques for enhanced STI outcomes.

STI faces several disruptive drivers of change including global changes i.e. socio-economic, geopolitical, scientific, rapid technological and environmental change, including the impacts of climate change (OECD 2018). These have profound implications for National Systems of Innovation (OECD 1997) as these drivers create challenges and opportunities. To illustrate, inter- and transdisciplinary knowledge is increasingly important, as research is becoming more data-driven (IEAG 2014, Department of Science and Technology 2019). This means that an open science approach is required to enable greater access to existing information (AOSP 2018). To support this, the move towards standardised machine-readable licence options for biodiversity data, enabled through the GBIF platform (GBIF 2016b), provided increased clarity for data users and publishers on the re-use of data for such inter and transdisciplinary research. Interestingly, the impact of the GBIF Research Infrastructure and its potential for STI can be seen in the research showcased in the GBIF science review (GBIF Secretariat 2019). Here, a steady increase in peer-reviewed publications from authors using GBIF-mediated data can be seen. This reflects an increase in use and impact of the data mobilised through the GBIF network in thematic areas including: invasive species, impacts of climate change, species conservation and protected areas, biodiversity and human health, food, farming and bio-fuels, ecosystem services, advancing biodiversity science, data management and data papers. It is also useful to note that use and impact statistics maybe even higher, as there is a profound cultural change that is needed in authors recognising the data providers as well as ensuring appropriate citation of the data (Escribano et al. 2018).

One of the mechanisms for increased innovation is an increase in publication output (OECD 2007, Inglesi-lotz and Pouris 2018). In the King Report (King 2004) evaluating the scientific impact of nations, citations were used as a proxy for science and technology intensity. Here, a correlation was drawn between scientific output and wealth intensity. Thus, if countries want to improve STI outputs and increase economic growth, they need to increase their investment in research and development. This will be especially relevant for sustainable development agenda and for research that supports socio-economically relevant areas, such as climate change, food security and human health.

The Heads of Delegation (HoD) to the GBIF Governing Board which are closely positioned to support governmental decision-making, have an instrumental strategic role in creating or supporting buy-in nationally, to strengthen efforts to grow biodiversity informatics as a field of science and, in turn, support STI. This includes generating/supporting buy-in with: national research foundations, funding agencies and policy/decision-makers. The key objectives here would be to support initiatives to establish Centres to develop BDI as a field of science. This includes funding to support Research Chairs and high-level academic expertise to catalyse BDI research and train the next generation of biodiversity informatics scientists and data scientists. The outcomes of such Centres will be established scientific research agendas, an increase in Honours, MSc and PhD postgraduate students in Biodiversity Informatics, engagement by the national community in a globally-emerging field of scientific research, as well as an increase in academic publications. It will also be important that the research agendas endeavour to support the SDG’s and science-policy efforts, such as IPBES, as well as other strategic priorities connected with human well-being.

HoDs can also further support the Nodes in the identification/development of strategic partnerships that can drive activities towards enhanced efforts in improving capacity in biodiversity informatics. This maybe for the purpose of implementing the curriculum at universities or for work-based training. HoDs may have existing bilateral agreements with countries or have access to other vehicles i.e. European Commission's Horizon 2020 and Horizon Europe 2021 that can be tapped into. Here, efforts should be made to explore and use existing agreements or establish new efforts to secure funding to support BDI activities.

Other potential strategic partnerships with regional bodies should also be explored, including with ministerial bodies, scientific or regional tertiary bodies. In Africa, this may include the African Union, the African Academy of Science, the African Research Universities Alliance (ARUA) and the Southern African Regional Universities Association (SARUA), respectively. In Europe, it may be European Union, the European Academy of Sciences and, in North America, the National Science Foundation.

**What does a biodiversity informatics curriculum mean for Node Managers**?

An established curriculum, with a set of teaching resources will enable the roll-out of a multitude of different BDI level courses, which can be offered at institutions or as a part of university level course. Such a curriculum will provide the basis for capacity building/enhancement in biodiversity informatics for students and work-based professionals at institutions and universities globally.

This survey sets out to explore the potential of the Nodes network to support training and teaching in BDI, as this area of work is increasingly being recognised as important for further formal development through professional biodiversity informatics networks, such as TDWG (TDWG 2019) and the GBIF Strategic plan and annual implementation workplan (GBIF 2019c). Sixty percent of Node Managers and Node staff expressed a willingness to mentor or train students/professionals and were prepared to be recruited or commissioned as part of a curriculum programme. They are, therefore, an excellent resource as potential trainers in a variety of training modules including biodiversity informatics, ICT development, data analysis, GIS, data capture, data cleaning and data publishing.

A list of URL’s containing training information and resources has also been provided which can be reviewed and re-used in the development of training and capacity development strategies by other Nodes or organisations. This can also be expanded upon in further studies. It would also be helpful to develop a more formal list of trainers, upon redistribution of the survey, who may be willing to fully support the training and teaching efforts of the BDI curriculum, as well as to support capacity development activities of Centres for Biodiversity Informatics. Here a similar process, like the GBIF Open Data Ambassadors concept (GBIF 2019e) can be put in place to secure further training expertise. The Carpentries network, which is a global network of trainers and mentors, teaching foundational data science and coding skills, should also be further explored to build and support this curriculum. The Carpentries also use a free, existing software platform to manage all of the curriculum that is open to the entire world to help develop and use.

It is noteworthy to mention that, since 2016, the GBIF Secretariat has actively been working on the development of curriculum components/modules, to support the Biodiversity Information for Development (BID) programme, aimed at the African, Caribbean and Pacific Regions. These reusable online modules will have systemic impact for the BDI curriculum teaching and training as a whole. The BID programme's focus was on building capacity for the publication and use of data, for the making of decisions in conservation and natural resource management. The curriculum included two courses: data mobilisation (GBIF 2016a, GBIF 2019d, GBIF 2017a, GBIF 2017c) and data use for decision-making GBIF 2019b). In 2019, a third training course was developed and implemented with a focus on curriculum relevant for capacity enhancement of Node Managers (GBIF 2019a). Here, four key areas for content development were identified to support node managers to effectively position their Nodes in the broader institutional landscape. These areas include the development of strategic plans and implementation plans that align with the GBIF strategic plan (2017-2021), to identify and engage key stakeholders to grow networks, organising training for workshops and developing re-usable training material and resourcing and collaborative project writing. All these courses bring together components from a wide range of disciplines and build on the knowledge within the GBIF network, with a number of Node Managers playing an active role as trainers and mentors in the roll-out of BID training events (GBIF 2020). The materials, developed in the context of the BID programme, will be made openly available to the wider biodiversity community. This can also be adapted for a more academic, university level course. An example of this is the data carpentries module (https://datacarpentry.org/semester-biology/).

Efforts, such as the GBIF Young Researchers Award and the GBIF Ebbe Nielson Prize, are also mechanisms to foster innovative research in biodiversity informatics and has the potential to raise the visibility of biodiversity informatics as a field of science at the national level. Here, Nodes play a critical role in galvanising the national community around the value of biodiversity informatics and can use the awards as a tool for awareness-raising of this new area of science.

## Conclusions

GBIF plays a critical role in a long data-science-policy value chain, where both the technological biodiversity informatics infrastructure, as well as the broad network of Participant Nodes and Partners, play a key enabling role towards growing biodiversity informatics as a field of science, through its capacity-building efforts. It has been identified that Biodiversity Informatics should be a part of the formal curricula at universities and here, GBIF could play a critical role, as an enabler, through the provision of training materials and content to support this field of work, as well as the network of Nodes and mentors, that have relevant expertise that can support the teaching and training for components of this field of science. It is imperative to harness the value of the GBIF Nodes network and strategic partnerships towards the best possible outcome, by ensuring that biodiversity informatics supports the best possible science and addresses global issues of significance, including global change and the bioeconomy. In the development of this field of biodiversity informatics science, efforts should be made towards maximising the use and relevance of revolutionary technologies i.e. mobile, robotics and big data and ensure that research infrastructures respond to societal demands and key policy directives i.e. the CBD, IPBES, UNFCCC, to conserve the Planet's precious biodiversity.

## Supplementary Material

3B90226F-BDBA-5B1F-9E93-4AE6FDD8AF21 10.3897/BDJ.9.e68010.suppl1 Supplementary material 1 Biodiversity Informatics Survey Data type Document - Survey Responses Brief description Document includes the survey questions and responses to the BDI session at the 2015 GBIF Nodes Meeting File: oo_576611.docx https://binary.pensoft.net/file/576611 Parker-Allie et al.

## Figures and Tables

**Table 1.** List of Uniform Resource Locators (URLs) providing training and dissemination information including events, activities and relevant resources of Nodes.

**URL**	**GBIF Node / Hosting Institutions**
1. https://drive.google.com/folderview?id=0B-WDk-H2QjRKfk9leWxPTGtLRTZvdjkwNzNjNWZMc1hzeHUzUWNFVDlhdDBTRTA3akMxNkE&usp=sharing	Species 2000, Naturalis Biodiversity Centre, (GBIF Participant Organisation)
2. http://www.gbif.es/en/formacion_ppal.php	GBIF-Spain; Real Jardín Botánico – CSIC (Higher Council of Scientific Research);
3. http://www.nabismaster.org/BL7035.php	Nordic Academy of Biodiversity and Systematic Studies - A collaboration between universities including: Norwegian University of Science and Technology, University of Oslo, Lund University, Stockholm University, University of Gothenburg, Uppsala University, University of Copenhagen
4. http://www.gbif.de/evertebrata2/zoologie_portal	GBIF Germany
5. http://www.gbif.no/events/	GBIF Norway, Natural History Museum, University of Oslo
6. https://sibcolombia.net/formacion/	GBIF Columbia, Instituto de Investigación de Recursos Biológicos Alexander von Humboldt
7. http://chm.aseanbiodiversity.org/thws/	Asean Centre for Biodiversity
8. http://www.gbif.fr/page/infos/formations	GBIF France; Muséum National d'Histoire Naturelle
9. http://www.rebioma.net	Wildlife Conservation Society Madagascar
10. https://www.gbif.pt/formacao	GBIF Portugal, Higher Institute of Agronomy, University of Lisbon
11. http://www.sibbr.gov.br and https://sibbr.gov.br/page/videos-tutoriais.html	GBIF Brazil, Ministry of Science, Technology, Innovation and Communication
12. http://www.gbifbenin.org/node/118	GBIF Benin, University of Abomey-Calavi
13. www.conabio.gob.mx	GBIF Mexico, The National Commission for the Knowledge and Use of Biodiversity (CONABIO)
14. http://vertnet.org/resources/workshops.html	VertNet is a collaborative project with core team members from Universities of California, Colorado, Kansas and Tulane and partners from a range of biodiversity projects.
15. www.danbif.dk	GBIF Node Denmark, University of Copenhagen
16. http://www.natureserve.org/conservation-tools/training	NatureServe (GBIF Participant Organisation)
17. http://biodiversityadvisor.sanbi.org/ http://biodiversityadvisor.sanbi.org/participation/sanbi-gbif-learning-network/ http://biodiversityadvisor.sanbi.org/participation/biodiversity-information-management-forum/biodiversity-information-management-forum-2/	GBIF South Africa (SANBI-GBIF), South African National Biodiversity Institute
18. http://taibif.tw/en/zh-gbif-training-materials-page https://www.gbif.jp/v2/activities/	GBIF Japan, Tokyo Metropolitan University

**Table 2.** Outcomes of the thematic breakout group and facilitated discussion at the 2015 GBIF Nodes Meeting, in Madagascar

**Needs Expressed by the Biodiversity Informatics Community**
1. Formal academic training in Biodiversity Informatics is critical to move from the short course nature of work-based training.
2. A prioritised list of training for work-based professionals is needed.
3. Training should be holistic, sequential and modular, speaking to the needs of different countries, which are at different stages of development.
4. There was a large interest in implementing e-learning platforms.
5. Resources are required to facilitate the interchange of high-level academic expertise between countries. This includes academics, such as professors.
**Recommendations and Outcomes from the Thematic Breakout Group**
1. Develop a GBIF-TDWG interest group, to support the curriculum. The Global Nodes Chair and a GBIF Secretariat representative for the training portfolio, should take the connection/process forward.
2. The survey should be redistributed to capture information on work–based training and academic teaching needs globally, as well as to capture the resources/materials and expertise. The survey time should be extended as well as the target audience. The survey should be redistributed to the Nodes for wider input and should be distributed globally, including in Biodiversity Information Standards (TDWG) group. (This was not conducted as part of the results and discussion of this study and can be addressed subsequently).
3. There are a number of existing resources and content already available to support Biodiversity Informatics curricula. A list of these training resources should be developed further. A GBIF task group should be developed to take this forward. The list should be dynamic and a platform should be identified to upload and maintain this list.
4. It was identified that a standard modular curriculum should be developed, which integrates all the existing curricula that already exists, as well as the results of the survey, as a reference for new ones.

**Table 3.** The Initial Working Biodiversity Informatics Curriculum

**Knowledge / Data Generation**	**Biodiversity Information Management** **(Post Data Capture)**	**Data Use and Application**
Introduction to Biodiversity and Data	Databases and their Design	Statistical Analysis of Biodiversity Data
Basics of Taxonomy, Taxonomic Databases, checklists	Programming/Scripting/Software Engineering	Species and Ecosystem Assessment
Biodiversity Data Capture and Data Quality Enrichment (e.g. Geo-referencing), Data Mobilisation	Biodiversity Data Assessment and Cleaning	Ecological Niche Modelling
Science Methods	Biodiversity Data Standards, Publishing and Licensing, Data policy and frameworks	Conservation Planning
Genetics and Molecular Data Generation	Geographic Information Systems (GIS)	Public Health Applications
Global Change Ecology (Climate, Invasive Aliens, Natural Resources)		Data-Science-Policy Interface
		Building Biodiversity Informatics Institutions

## Footnotes

*1
https://www.gbif.org/news/2ggIFqrxre4im4oSMeMmA2/gbif-formalizes-collaboration-with-biodiversity-assessment-platform

*2
https://www.ipbes.net/assessment-reports

*3
https://www.bipindicators.net/indicators/growth-in-species-occurrence-records-accessible-through-gbif

*4
http://biodiversityadvisor.sanbi.org/wp-content/uploads/2016/05/Strategy-for-Developing-a-Centre-for-Biodiversity-Information_11May2016.pdf

*5
https://www.tdwg.org/community/curriculum/

*6
http://biodiversity-informatics-training.org/

*7
https://www.gbif.org/project/82649/biodiversity-informatics-cookbook
