# Supplementary material for: Towards a Post-Graduate Level Curriculum for Biodiversity Informatics. Perspectives from the Global Biodiversity Information Facility (GBIF) Community
Source: Biodivers Data J. 2021 Oct 7;9:e68010. doi: 10.3897/BDJ.9.e68010 (PMC8516826; doi:10.3897/BDJ.9.e68010)
Supplement: Supplementary material 1 — Biodiversity Informatics Survey [file bdj-09-e68010-s001.docx]

**Supplementary Information**

# Annexure A. GBIF Survey Results (October 2015): Towards a Curriculum for Biodiversity Informatics

A survey was distributed to the GBIF Interest Group, prior to the 13^th^ GBIF Nodes Meeting (2015). It was intended to get an idea of the current level of activity in the GBIF network in connection with work-based training and/or academic teaching (at universities) in the field of biodiversity informatics.

It was planned to get an overview of Node Managers already engaged in developing course curricula as part of academic processes or providing training, and whether participants would be willing to share resources or enter into collaborations using the means offered by GBIF Nodes globally.

The survey had two parts. The first part contained six quick questions and Nodes were invited to answer them. The second part included ten questions with a higher level of detail, and participants were invited to respond to those questions as well.

# PART ONE: SHORT QUESTIONS RELATING TO THE NODE

**Question 1. Please select the items that describe the training and capacity enhancement activities organized by your Node**


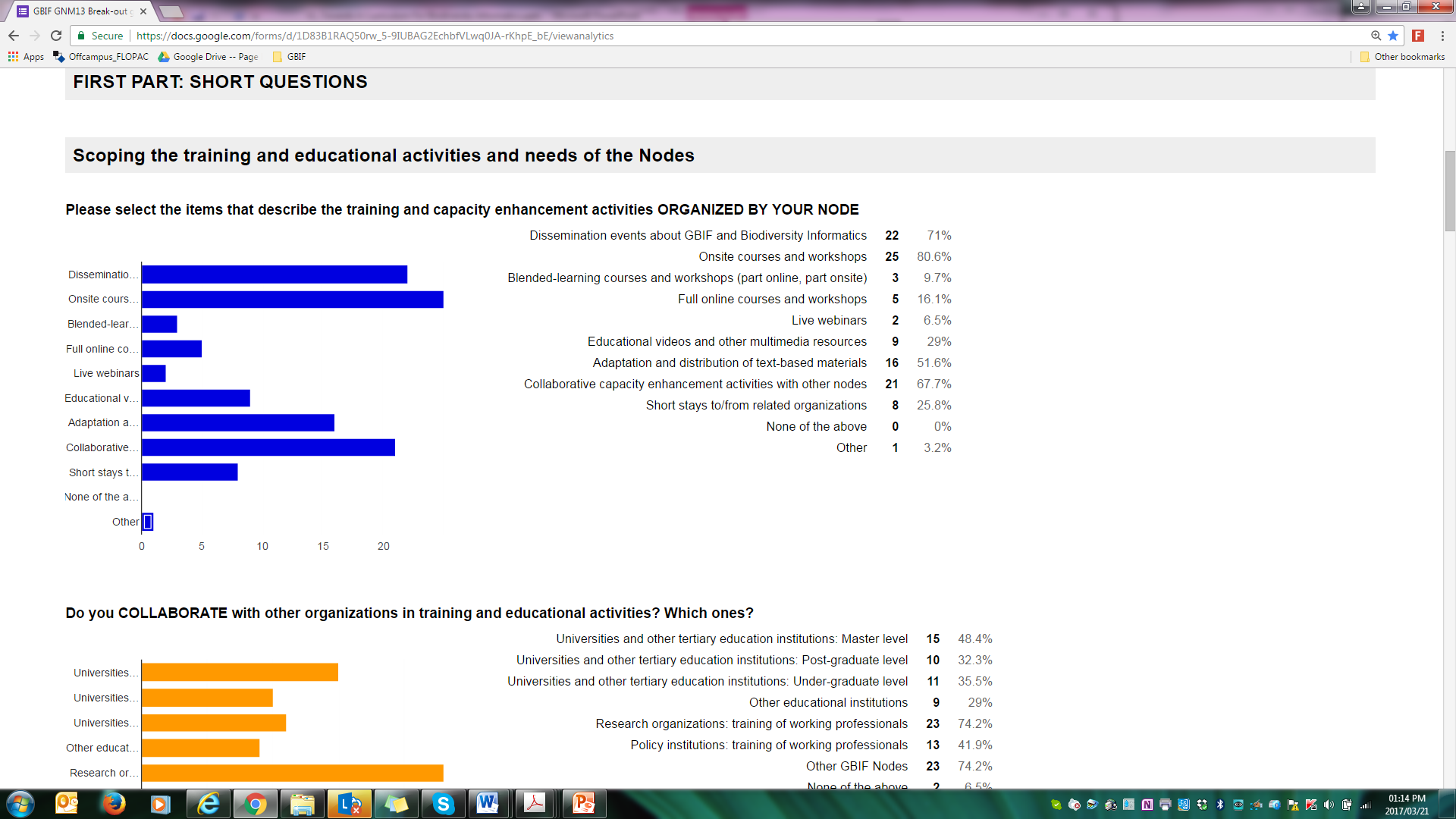
Answered: 31 Skipped: 0

| **Answer Choices** | **Responses** | **Percentage** |
| --- | --- | --- |
| Dissemination events about GBIF and Biodiversity Informatics | **22** | 71% |
| Onsite courses and workshops | **25** | 81% |
| Blended-learning courses and workshops (part online, part onsite) | **3** | 10% |
| Full online courses and workshops | **5** | 16% |
| Live webinars | **2** | 7% |
| Educational videos and other multimedia resources | **9** | 29% |
| Adaptation and distribution of text-based materials | **16** | 52% |
| Collaborative capacity enhancement activities with other nodes | **21** | 68% |
| Short stays to/from related organizations | **8** | 26% |
| None of the above | **0** | 0% |
| Other | **1** | 3% |

**Question 2. Which TOPICS are you currently covering in your training and dissemination activities?**

Answered: 31 Skipped: 0


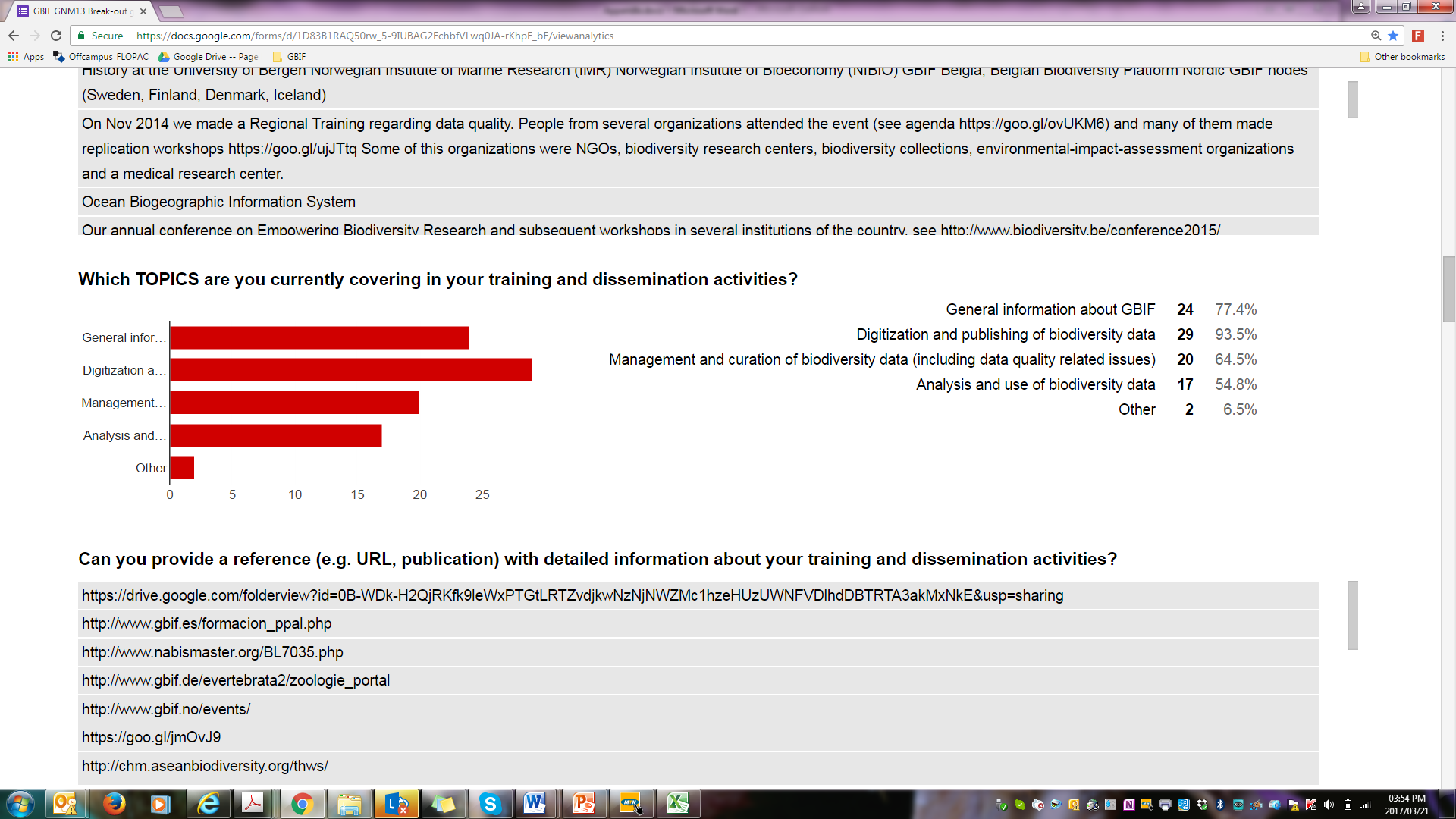


| **Answer Choices** | **Responses** | **Percentage** |
| --- | --- | --- |
| General information about GBIF | **24** | 77% |
| Digitization and publishing of biodiversity data | **29** | 94% |
| Management and curation of biodiversity data (including data quality related issues) | **20** | 65% |
| Analysis and use of biodiversity data | **17** | 55% |
| Other | **2** | 7% |

**Question 3. Do you COLLABORATE with other organizations in training and educational activities? Which ones?**

Answered: 31 Skipped: 0


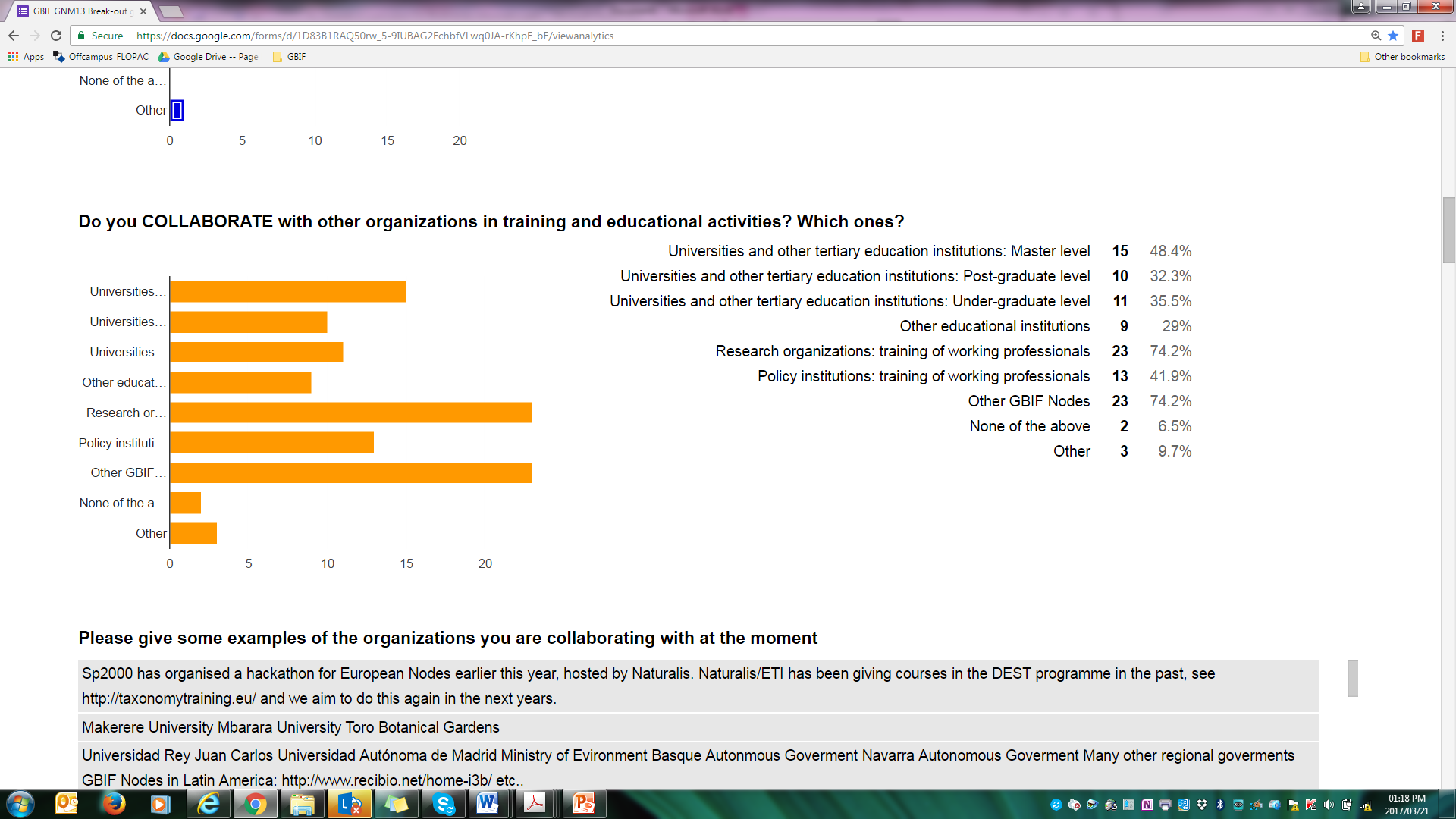


| **Answer Choices** | **Responses** | **Percentage** |
| --- | --- | --- |
| Universities and other tertiary education institutions: Master level | **15** | 48% |
| Universities and other tertiary education institutions: Post-graduate level | **10** | 32% |
| Universities and other tertiary education institutions: Under-graduate level | **11** | 36% |
| Other educational institutions | **9** | 29% |
| Research organizations: training of working professionals | **23** | 74% |
| Policy institutions: training of working professionals | **13** | 42% |
| Other GBIF Nodes | **23** | 74% |
| None of the above | **2** | 7% |
| Other | **3** | 10% |

**Question 4. Please give some examples of the organizations you are collaborating with at the moment**

Answered: 26 Skipped: 5

| **Number** | **Answer** | | **Timestamp** |
| --- | --- | --- | --- |
| 1. Sp2000 has organised a hackathon for European Nodes earlier this year, hosted by Naturalis. Naturalis/ETI has been giving courses in the DEST programme in the past, see http://taxonomytraining.eu/ and we aim to do this again in the next years. | | 9/15/2015 13:39:58 | |
| 1. Makerere University, Mbarara University, Toro Botanical Gardens | | 9/16/2015 7:07:38 | |
| 1. Universidad Rey Juan Carlos and Universidad Autónoma de Madrid, Ministry of Environment, Basque Autonomous Government and Navarra Autonomous Government. Many other regional governments. GBIF Nodes in Latin America: http://www.recibio.net/home-i3b/ etc. | | 9/16/2015 8:41:33 | |
| 1. Ministère de l'enseignement supérieur et de la recherche scienifique, WCS / Rebioma, University of Mahajanga, University of Tuléar, University of Antananarivo, University of Tamatave, University of Fianarantsoa, PBZT, FOFIFA. | | 9/16/2015 9:52:50 | |
| 1. The nine Nordic universities collaborating on the NABiS master programme in Biodiversity and Systematics (http://www.nabismaster.org/). Also Stockholm University in closer collaboration. NGO’s supporting "Science Festivals" etc.). | | 9/16/2015 15:33:18 | |
| 1. gbif-mykologie.de | | 9/16/2015 15:53:35 | |
| 1. Our host the Natural History Museum, University of Oslo University of Oslo (UiO.no) Norwegian Institute for Nature Research (NINA.no) NTNU University Museum, Trondheim Natural History at the University of Bergen Norwegian Institute of Marine Research (IMR) Norwegian Institute of Bioeconomy (NIBIO), GBIF Belgia, Belgian Biodiversity Platform Nordic, GBIF nodes (Sweden, Finland, Denmark, Iceland) | | 9/16/2015 18:26:00 | |
| 1. On Nov 2014 we conducted a regional training workshop in data quality. People from several organizations attended the event (see agenda https://goo.gl/ovUKM6) and many of them subsequently conducted replication workshops (<https://goo.gl/ujJTtq>). Some of these organizations were NGOs, biodiversity research centres, biodiversity collections, environmental-impact-assessment organizations and a medical research centre. | | 9/16/2015 19:14:40 | |
| 1. Ocean Biogeographic Information System | | 9/17/2015 4:08:37 | |
| 1. Our annual conference on Empowering Biodiversity Research and subsequent workshops in several institutions of the country. see http://www.biodiversity.be/conference2015/ | | 9/17/2015 11:21:05 | |
| 1. Follow-up of previous GBIF Mentoring programmes with Togo and Mauritania (helping them gaining autonomy in data publication) - Preparation of the GB22 nodes training event | | 9/17/2015 14:04:23 | |
| 1. We presented the activities and GBIF portal to the students of the master UE Biodiversity Informatics of University Pierre et Marie Curie Paris 6. We organised a training event for researchers with the FRB: Foundation for Research on Biodiversity, a platform for the different scientific players and stakeholders in society concerned with biodiversity. It was created in 2008, following the Grenelle Environment Forum, with the support of the ministries for research and ecology and eight public research establishments, joined by LVMH in 2014. http://www.gbif.fr/formation_septembre2015/ | | 9/18/2015 16:31:38 | |
| 1. REPC- Réseau des Educateurs Professionnels de la Conservation: http://www.amnh.org/our-research/center-for-biodiversity-conservation/capacity-development/network-of-conservation-educators-and-practitioners-ncep or http://www.conservation.org/global/madagascar/nos-activites/repc/Pages/repcdetail.aspx | | 9/22/2015 10:14:11 | |
| 1. Ministry of sustainable development and infrastructures, Administration of nature and forests, Biological stations, Nature parks | | 9/22/2015 10:57:20 | |
| 1. We have worked together with GBIF nodes in the region such as TanBIF and UgaBIF to conduct introductory trainings on biodiversity intended for conservation practitioners and researchers in the region. In the future, we are considering partnership with projects like IDigBio to improve the digitization schemes we are undertaking in herbaria in the region. | | 9/22/2015 12:03:20 | |
| 1. Workshop on Specify 6 - Herbarium of Luanda Workshop on Specify 6 - SiBBr CIBIO/InBio, Associate Laboratory, University of Porto | | 9/22/2015 12:41:54 | |
| 1. University of Nouakchott, National Parks and Oceanographic Institute | | 9/22/2015 13:34:08 | |
| 1. University of Kansas, Biodiversity Institute | | 9/22/2015 16:07:15 | |
| 1. University of Kansas, University of Florida, UC Berkeley, iDigBio | | 9/22/2015 18:01:03 | |
| 1. UNLP, Universidad Nacional de La Plata (National University of La Plata) | | 9/22/2015 18:43:32 | |
| 1. GBIF Secretariat - GBIF Belgium - TDWG - University of Kansas | | 9/22/2015 21:12:41 | |
| 1. We collaborate with our member programs that are located across the U.S. and Canada located in state agencies, universities and NGOs. | | 9/23/2015 23:32:33 | |
| 1. Universities, museums, research organisations, provincial organisations, government departments | | 9/26/2015 17:48:23 | |
| 1. We've given scattered lectures about biodiversity informatics on different levels/university courses, both for our parent organization University of Helsinki and also for Scandinavian and Russian students as part of a Norwegian ForBio: Research School in Biosystematics (www.forbio.uio.no/) | | 9/29/2015 16:03:09 | |
| 1. Hosting training workshops for projects sponsored by Forest Bureau and Ministry of Science and Technology. | | 9/30/2015 3:03:03 | |
| 1. I take some class for undergraduate class in 2 universities as part time lecture | | 10/2/2015 16:37:02 | |

**Question 5. Can you provide a reference (e.g. URL, publication) with detailed information about your training and dissemination activities?**

Answered: 21 Skipped: 10

| **Number** | | **Answer** | | **Timestamp** | |
| --- | --- | --- | --- | --- | --- |
| 1 | | <https://drive.google.com/folderview?id=0B-WDk-H2QjRKfk9leWxPTGtLRTZvdjkwNzNjNWZMc1hzeHUzUWNFVDlhdDBTRTA3akMxNkE&usp=sharing> | | 9/15/2015 13:39:58 | |
| 2 | | <http://www.gbif.es/formacion_ppal.php> | | 9/16/2015 8:41:33 | |
| 3 | | <http://www.nabismaster.org/BL7035.php> | | 9/16/2015 15:33:18 | |
| 4 | | <http://www.gbif.de/evertebrata2/zoologie_portal> | | 9/16/2015 15:53:35 | |
| 5 | | <http://www.gbif.no/events/> | | 9/16/2015 18:26:00 | |
| 6 | | <https://goo.gl/jmOvJ9> | | 9/16/2015 19:14:40 | |
| 7 | | <http://chm.aseanbiodiversity.org/thws/> | | 9/17/2015 4:08:37 | |
| 8 | | <http://www.gbif.fr/page/infos/formations> | | 9/18/2015 16:31:38 | |
| 9 | | [http://www.rebioma.net](http://www.rebioma.net/) | | 9/22/2015 10:14:11 | |
| 10 | | <http://www.iict.pt/specify/> | | 9/22/2015 12:41:54 | |
| 11 | | <http://www.sibbr.gov.br/internal/?area=comunicacao&subarea=agenda> | | 9/22/2015 16:01:11 | |
| 12 | | Reports are available in GBIF secretariat and JRS Biodiversity Foundation | | 9/22/2015 16:07:15 | |
| 13 | | [www.conabio.gob.mx](http://www.conabio.gob.mx/) | | 9/22/2015 17:51:20 | |
| 14 | | <http://vertnet.org/resources/workshops.html> | | 9/22/2015 18:01:03 | |
| 15 | | Is not on line | | 9/22/2015 18:43:32 | |
| 16 | | not online | | 9/22/2015 21:12:41 | |
| 17 | | [www.danbif.dk](http://www.danbif.dk/) | | 9/23/2015 15:29:33 | |
| 18 | | <http://www.natureserve.org/conservation-tools/training> | | 9/23/2015 23:32:33 | |
| 19 | | <http://biodiversityadvisor.sanbi.org/> | | 9/26/2015 17:48:23 | |
| 20 | | http://taibif.tw/en/zh-gbif-training-materials-page","http://taibif.tw/en/zh-gbif-training-materials-page | | 9/30/2015 3:03:03 | |
| 21 | | General information about GBIF, Digitization and publishing of biodiversity data, Analysis and use of biodiversity data | | 10/2/2015 16:37:02 | |

**Q6. Does your country/organization/BIF have any emerging training INTERESTS or NEEDS that you would like to cover in the future? Please describe them. If you already have plans on how to cover those needs, please describe them too.**

Answered: 22 Skipped: 9

| **Number** | | **Answer** | | **Timestamp** |
| --- | --- | --- | --- | --- |
| 1. SP2000 aims to create a training event for creating and maintaining species checklists. Naturalis aims to create training materials for creating species information systems with Linnaeus NG (training materials planned for spring 2016). Naturalis also aims to give training in providing data sources to GBIF with the Netherlands Biodiversity API (NBA). | | 9/15/2015 13:39:58 | | |
| 1. data analysis, niche modelling, large data mining, IPT and Data paper | | 9/16/2015 7:07:38 | | |
| 1. Just one over the rest: Formal training in Biodiversity Informatics; we need to put that in the University, to mainstream it. We have some advances and collaborations with universities, but these are too few, progress is too slow. What we do as a GBIF Node is (I think) remarkable, but it is cottage industry; that helps but does not make an impact in the next generation of biologists and other environmental professionals. | | 9/16/2015 8:41:33 | | |
| 1. In this time I want to just know how to digitalize biodiversity data and how to use them and to share them with my colleagues. | | 9/16/2015 9:52:50 | | |
| 1. Spread Biodiversity data | | 9/16/2015 15:13:25 | | |
| 1. Expected to jointly with Stockholm University develop undergraduate education (mostly on-site) in Biodiversity Informatics. Also we will collaborate on all the above mentioned aspects within a new national infrastructure for Biodiversity and Ecosystems research in Sweden. Videos, movies etc. already exist. | | 9/16/2015 15:33:18 | | |
| 1. Our node does not have the capacity for that (only part-time employee) | | 9/16/2015 15:53:35 | | |
| 1. Norwegian researchers working with monitoring of biodiversity, in particular form vegetation science, have approached GBIF Norway for assistance with a data management plan including long-term archiving and publishing of respective datasets. We plan a workshop on data publishing in Trondheim 29th and 30th October with focus on the new Event Core for Darwin Core archives. We have initiated contact with the Norwegian EUDAT node for exploring better models for long-term archiving of Norwegian biodiversity datasets including vegetation science datasets. | | 9/16/2015 18:26:00 | | |
| 1. One of the key features that we would like to further enhance is using and downloading occurrence data coming from GBIF. With this initiative we can focus on species conservation and gap analysis for ASEAN member states and this will be their baseline as monitoring species conservation. | | 9/17/2015 4:08:37 | | |
| 1. Online courses on Biodiversity Informatics: from basic data management up to more advanced topics. | | 9/17/2015 11:21:05 | | |
| 1. I have "personal/informal" plan to gradually create materials on the topic of "Data management for scientists" (normalization, data cleaning, basic database use, ...) | | 9/17/2015 14:04:23 | | |
| 1. Following the mentoring we had with Spain and Portugal in 2014, we are currently building our e-learning platform. We just recorded a training we made last week on data publication, data quality and data papers. We are interested in the development of collaboration with universities | | 9/18/2015 16:31:38 | | |
| 1. Organising online courses would be useful | | 9/22/2015 10:57:20 | | |
| 1. In the countries we operate in (mainly Rwanda, Burundi and Eastern DRC), there is urgent need for capacity building in topics related to data capture (digitization, geo-referencing, data cleaning, etc.) as well as data management and publishing (data standards, data analysis, etc.). | | 9/22/2015 12:03:20 | | |
| 1. Geo-referencing occurrences data | | 9/22/2015 16:01:11 | | |
| 1. In-depth training at master and PhD levels through a sound program to be settled in place in Benin | | 9/22/2015 16:07:15 | | |
| 1. Knowledge and management of biodiversity information (quantity of data added to the National System of Biological Data and others). Quantity of samples in biological collections. *Professional training. *System of Biodiversity Information (institutions collaborating on the portals, quantity of data and sharing resources). | | 9/22/2015 18:43:32 | | |
| 1. We are interested in offering more online training and in-depth workshops in areas related to species status assessments, species location mapping, climate change assessments, biodiversity monitoring, predictive distribution mapping, and integrated land-use planning. | | 9/23/2015 23:32:33 | | |
| 1. In South Africa we are looking at developing a Centre for Biodiversity Informatics. We are developing curricula at postgraduate level and working with Universities for implementation. | | 9/26/2015 17:48:23 | | |
| 1. We are interested in collaboration and developing BDI curriculum. So far, we have given lectures and plenary talks on subjects that were defined by organiser/funder. First we should get an agreement with our own university how to merge BDI into their Biodiversity & Ecology curriculum. | | 9/29/2015 16:03:09 | | |
| 1. "TaiBIF will focus on three main themes when hosting training workshops in the near future: 1) Promote open data license; 2) Provide tools/services (scientific name matching) to improve data quality; 3) Provide courses of reusing open data for biodiversity research and conservation." | | 9/30/2015 3:03:03 | | |
| 1. JBIF has a plan to publish the instructional text in Japanese language for undergraduate students. | | 10/2/2015 16:37:02 | | |

**Q7. Can you provide us with a bit of more detail about your activities?**

Answered: 31 Skipped: 0


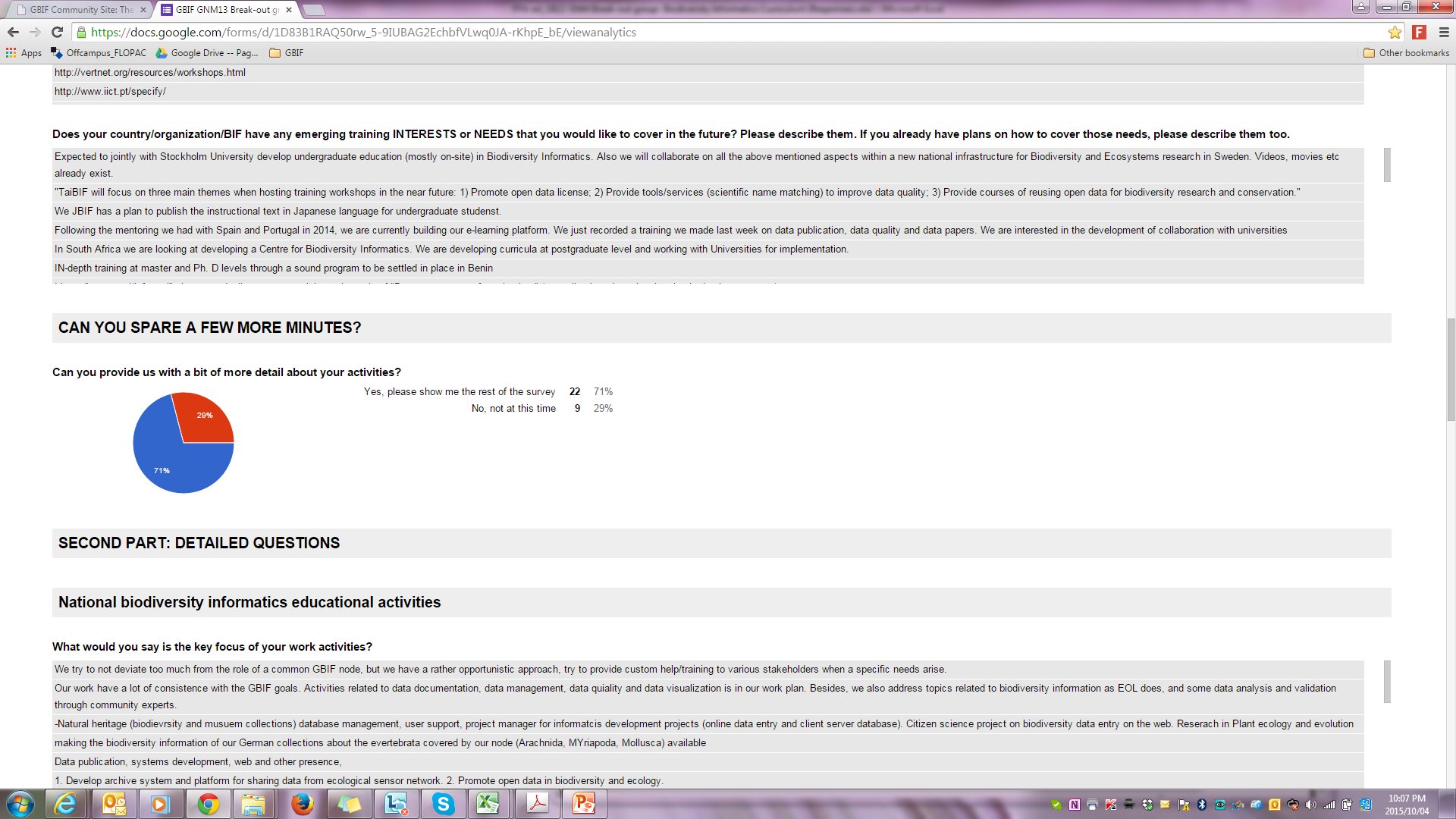


| **Answer Choices** | **Responses** | **Percentage** |
| --- | --- | --- |
| Yes, please show me the rest of the survey | **22** | 71% |
| No, not at this time | **9** | 29% |

# SECOND PART: DETAILED QUESTIONS

# NATIONAL BIODIVERSITY INFORMATICS ACADEMIC EDUCATIONAL ACTIVITIES

# Q8. What would you say is the key focus of your work activities?

Answered: 19 Skipped: 12

| **Number** | | | **Answer** | | **Timestamp** |  |
| --- | --- | --- | --- | --- | --- | --- |
|  | 1 |  | Making the Catalogue of Life available, Creating an ICT infrastructure for Naturalis and other Dutch organisations (Netherlands Biodiversity API and Naturalis BioPortal) |  | 9/15/2015 13:39:58 |  |
|  | 2 |  | Data use and digitisation |  | 9/16/2015 7:07:38 |  |
|  | 3 |  | We have a full-fledged training program involving at least 11 workshops a year. We do that with a focus on GBIF (and national) goals. We do not charge participants (and we do not cover any travel expense) but we handpick the participants on the basis of their potential to become GBIF data publishers, or because it is strategic to us for reaching "new" communities (e.g. marine biologists, land administrations, etc.).... it works |  | 9/16/2015 8:41:33 |  |
|  | 4 |  | Data publication, systems development, web and other presence, |  | 9/16/2015 15:33:18 |  |
|  | 5 |  | Making the biodiversity information of our German collections about the evertebrata covered by our node (Arachnida, Myriapoda, Mollusca) available |  | 9/16/2015 15:53:35 |  |
|  | 6 |  | Key focus is as for most nodes on providing a helpdesk for publishing Norwegian biodiversity data to GBIF. In addition, we offer support for the use of biodiversity data including data that can be accessed through GBIF. |  | 9/16/2015 18:26:00 |  |
|  | 7 |  | Our work has a lot of consistency with the GBIF goals. Activities related to data documentation, data management, data quality and data visualization is in our work plan. Besides, we also address topics related to biodiversity information as EOL does, and some data analysis and validation through community experts. |  | 9/16/2015 19:14:40 |  |
|  | 8 |  | Managing species occurrence data and other Bioinformatics related databases. |  | 9/17/2015 4:08:37 |  |
|  | 9 |  | Suggestion of appropriate tools to scientists/data holders. |  | 9/17/2015 11:21:05 |  |
|  | 10 | We try to not deviate too much from the role of a common GBIF node, but we have a rather opportunistic approach. We try to provide custom help/training to various stakeholders when specific needs arise. | | 9/17/2015 14:04:23 | | |
|  | 11 | Mobilization and data publication, data quality issues, trainings, development of a national portal, interactions at national level with ministries and other organisations dealing with biodiversity and support to francophone countries. We don't really deviate from the common GBIF node. | | 9/18/2015 16:31:38 | | |
|  | 12 | Natural heritage (biodiversity and museum collections) database management, user support, project manager for informatics development projects (online data entry and client server database). Citizen science project on biodiversity data entry on the web. Research in Plant ecology and evolution | | 9/22/2015 10:57:20 | | |
|  | 13 | At ARCOS we are interested in mobilizing environmental data including biodiversity data and analysing the data to influence decision making especially on development activities that affect freshwater and mountain ecosystems in areas we operate in. We would like to see various datasets produced by various stakeholders made available so that a regular accurate reporting on the environmental status of the region can become possible. | | 9/22/2015 12:03:20 | | |
|  | 14 |  | Collecting and digitizing data on Mauritanian Biodiversity |  | 9/22/2015 13:34:08 |  |
|  | 15 |  | Data mobilization, training, data uses to derive research products to inform decision |  | 9/22/2015 16:07:15 |  |
|  | 16 |  | My activities are quite broad. General Node Manager responsibilities, including engagement with stakeholders to share data, managing training workshops/workshops, coordinating annual Biodiversity Information Management Forum; and also responsible for managing an initiative towards developing a Centre for Biodiversity Information Management. |  | 9/26/2015 17:48:23 |  |
|  | 17 |  | My main responsibility at the moment is leading (and seeking more funding for) projects that aim at open data sharing and data interoperability. |  | 9/29/2015 16:03:09 |  |
|  | 18 |  | 1. Develop archive system and platform for sharing data from ecological sensor network.  2. Promote open data in biodiversity and ecology. |  | 9/30/2015 3:03:03 |  |
|  | 19 |  | How to initiate the Biodiversity Informatics for developing countries. |  | 10/2/2015 16:37:02 |  |

**Q9. Have you had any academic teaching experience or work-based experience as a trainer? Please provide details of this experience**

Answered: 19 Skipped: 12

| **Number** | **Answer** | **Date** |
| --- | --- | --- |

| 1 | Yes, giving biodiversity informatics course for post-docs, train the trainer’s session for nodes in East Africa (Tanzania, Uganda). | 9/15/2015 13:39:58 |
| --- | --- | --- |
| 2 | A little | 9/16/2015 8:41:33 |
| 3 | Yes. I am a teacher in some private university in Antananarivo. I teach and give information to the student concerning the biodiversity and de conservation of the Malagasy fauna and the flora, tourism, ecotourism and education for sustainable development. | 9/16/2015 9:52:50 |
| 4 | Being an associate professor in ecology, botany and teacher training, and having taught these subjects for 30 odd years before entering as GBIF Node Manager I do feel I have a bit of experience. Explicit BI training is restricted to the past 15 years. | 9/16/2015 15:33:18 |
| 5 | in undergraduate courses, more than 20 years ago | 9/16/2015 15:53:35 |
| 6 | The Norwegian node contributes to the university training at the museum. Examples include contribution to an annual master/Phd course on species distribution modelling; master/phd training on spatial data analysis and visualisation methods using a GIS. We contribute to master, Phd, and postdoc supervision and support including supervision, analysis and distribution modelling for crop wild relatives. I also have previous teaching experience from similar topics at the Nordic Genetic Resources Centre. | 9/16/2015 18:26:00 |
| 7 | Me, only during the GB20 at Berlín. My team have more experience organizing workshops and trainings to Spanish speaking audiences. | 9/16/2015 19:14:40 |
| 8 | Resource speaker on managing Biodiversity data. Standardization of Biodiversity data using Darwin-Core. | 9/17/2015 4:08:37 |
| 9 | Generic IT courses at undergraduate degree. | 9/17/2015 11:21:05 |
| 10 | Most experience was gained through participation to various biodiversity-related training and capacity-building events, for example: - GBIF Mentoring programme with Togo and Mauritania - One CABIN mission in Kinshasa - Trainer for events at GB18, GB20 and GB22 | 9/17/2015 14:04:23 |
| 11 | Only work-based experience : trainer during different events organised by GBIF France in France and Africa, and for the nodes training event in Buenos Aires | 9/18/2015 16:31:38 |
| 12 | Seminar on plant population biology and biodiversity data management at the University of Metz | 9/22/2015 10:57:20 |
| 13 | No | 9/22/2015 13:34:08 |
| 14 | During the training workshop I organized, I taught on data mobilization (data types, data publishing process, data representation to raise awareness of decision makers and potential data providers) | 9/22/2015 16:07:15 |
| 15 | I have had training from NCTC, but most of my experience comes from my own reading and working with others on designing and implementing training. | 9/23/2015 23:32:33 |
| 16 | No; i have done some lectures, presentations, outreach as a Node Manager including at GBIF meetings. I have good experience engaging with stakeholders and communities but have not done training in a specific work areas with students. | 9/26/2015 17:48:23 |
| 17 | I've given a few lectures and workshops mainly as part of projects that I've participated or lead. The professional workshops have mainly aimed at describing data interoperability issues with a framework called "Enterprise architecture". University lectures (PhD student level) were about data re-usability and open scientific data, data curation work flows, and other data management, but not going to the actual analyses. | 9/29/2015 16:03:09 |
| 18 | Yes. I taught courses of general ecology, application of GIS/remote sensing in ecology, and landscape ecology during 2005-2009. I also organized regional training workshops for IPT2, ecological information management, ecological sensor network, and soundscape monitoring during 2010-2015. | 9/30/2015 3:03:03 |
| 19 | No but I sometime mentioned the Biodiversity Informatics in the lecture time in university and open seminar. | 10/2/2015 16:37:02 |

**Q10. Are you engaged or aware of any institutions/university in your country that is offering a Biodiversity Informatics Course, undergraduate degree or Postgraduate degree? If you are engaged in any of these activities, please indicate in which capacity**

Answered: 18 Skipped: 13

| \| **Number** \| **Answer** \| **Timestamp** \| \| \| --- \| --- \| --- \| --- \| \| 1 \| Not outside Naturalis. \| 9/15/2015 13:39:58 \| \| 2 \| Yes, Makerere University- Industry based expert \| 9/16/2015 7:07:38 \| \| 3 \| Yes, two master courses, teaching specific subjects \| 9/16/2015 8:41:33 \| \| 4 \| At this time, no, but I hope later \| 9/16/2015 9:52:50 \| \| 5 \| As mentioned in the first part of the survey I know most Swedish universities are open to, or already do teach BI to some (basic) extent, but normally not at undergraduate level which I think is the most critical part of bringing the topic to students. I lend myself to those interested. \| 9/16/2015 15:33:18 \| \| 6 \| I am aware of, but have no direct involvement \| 9/16/2015 15:53:35 \| \| \| 7 \| I am not aware of any dedicated biodiversity informatics curriculum offered in Norway. However, biodiversity informatics is starting to be integrated in university training - including two recent academic teaching positions (associate professorships) on biodiversity informatics at the NTNU university in Trondheim and the University of Bergen. \| 9/16/2015 18:26:00 \| \| \| 8 \| Not currently, but we are working with at least 4 universities that are willing to adopt courses on this topic \| 9/16/2015 19:14:40 \| \| \| 9 \| University of the Philippines \| 9/17/2015 4:08:37 \| \| \| 10 \| Yes, we are included in such activities at UGent and ULB. We are invited there to give training on Biodiversity data publication. \| 9/17/2015 11:21:05 \| \| \| 11 \| Not personally other people in our Node are (André Heughebaert and Dimitri Brosens, for example) \| 9/17/2015 14:04:23 \| \| \| 12 \| Regine Vigne, our scientific coordinator at GBIF France is Professor at the University Paris 6 in systematic and Biodiversity Informatics. We quickly presented GBIF in this course but we want to do it more consequently. \| 9/18/2015 16:31:38 \| \| \| 13 \| In the countries we operate in, biodiversity bioinformatics is a field still in its infancy. The topic is mentioned in various courses at academia here just as one of the emergent fields in biology and biogeography but no specific course deals specifically with the topic. \| 9/22/2015 12:03:20 \| \| \| 14 \| No \| 9/22/2015 13:34:08 \| \| \| 15 \| I am actually promoting BI in my university so that in the years coming I will be able to set master and Ph. D programs \| 9/22/2015 16:07:15 \| \| \| 16 \| Yes -University of Western Cape currently offers an BSc Honours elective module (7-8 weeks) in Biodiversity Information Management, which is part of a SANBI lead initiative. \| 9/26/2015 17:48:23 \| \| \| 17 \| Currently, no. We plan to do so in the near future. \| 9/30/2015 3:03:03 \| \| \| 18 \| none \| 10/2/2015 16:37:02 \| \| |
| --- | --- | --- | --- | --- | --- | --- | --- | --- | --- | --- | --- | --- | --- | --- | --- | --- | --- | --- | --- | --- | --- | --- | --- | --- | --- | --- | --- | --- | --- | --- | --- | --- | --- | --- | --- | --- | --- | --- | --- | --- | --- | --- | --- | --- | --- | --- | --- | --- | --- | --- | --- | --- | --- | --- | --- | --- | --- | --- | --- | --- | --- | --- | --- | --- | --- | --- | --- | --- | --- | --- | --- |

**Q11. Would you be willing to make your course content openly available to support the further rollout and uptake of BI as a field of science?**

Answered: 21 Skipped: 10


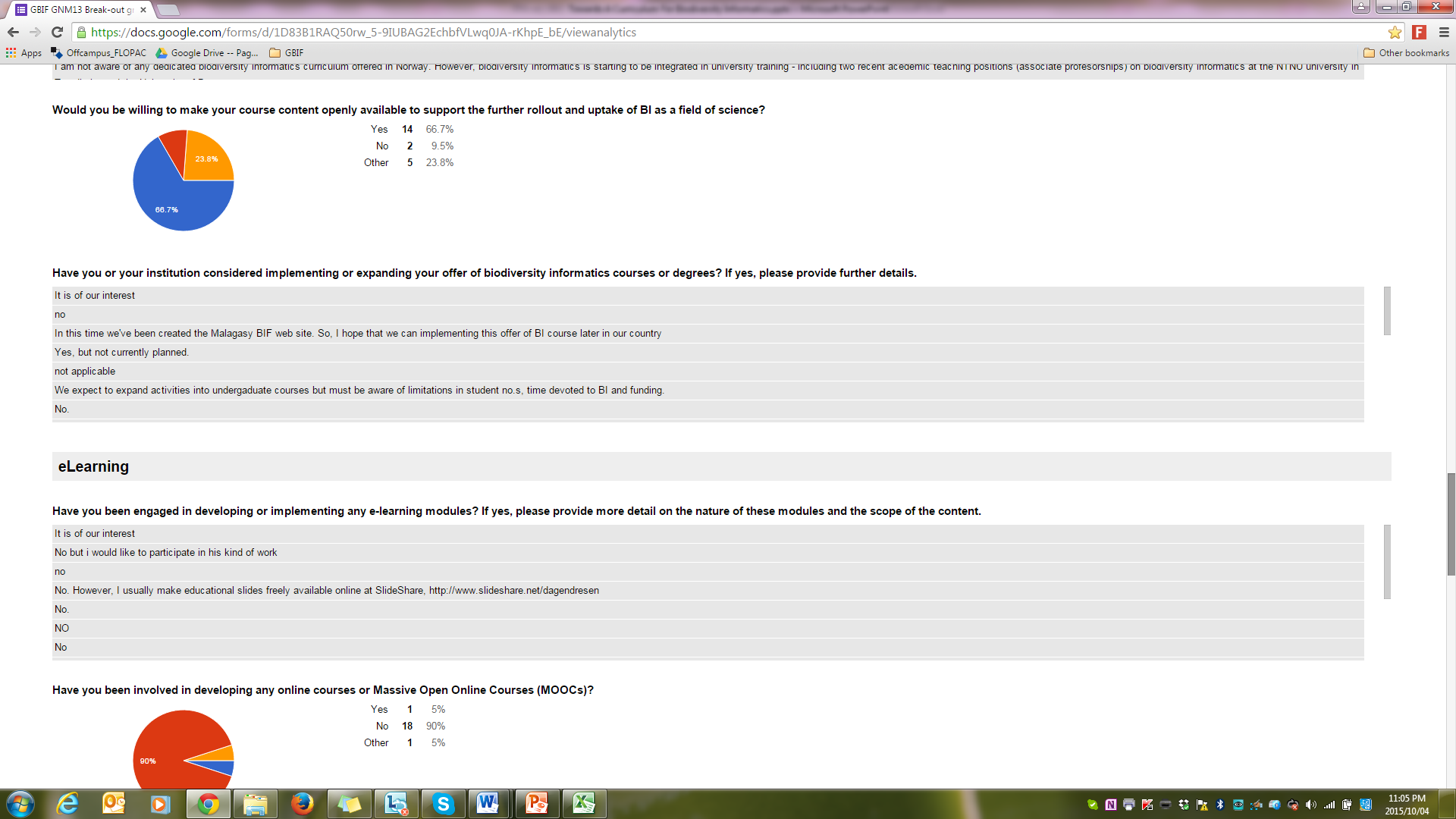


| **Answer Choices** | **Responses** | **Percentage** |
| --- | --- | --- |
| Yes | **14** | 67% |
| No | **2** | 10% |
| Other | **5** | 24% |

**Q12. Have you or your institution considered implementing or expanding your offer of biodiversity informatics courses or degrees? If yes, please provide further details**

Answered: 17 Skipped: 15

| \| Number \| Answer \| Timestamp \| \| --- \| --- \| --- \| \| 1 \| Yes, but not currently planned. \| 9/15/2015 13:39:58 \| \| 2 \| Not yet \| 9/16/2015 7:07:38 \| \| 3 \| I do not think we could do anything else beyond what we are currently doing under the current circumstances and budget... As I mentioned before the future is to transfer those activities to universities \| 9/16/2015 8:41:33 \| \| 4 \| In this time we've been created the Malagasy BIF web site. So, I hope that we can implementing this offer of BI course later in our country \| 9/16/2015 9:52:50 \| \| 5 \| We expect to expand activities into undergraduate courses but must be aware of limitations in student numbers, time devoted to Biodiversity informatics and funding. \| 9/16/2015 15:33:18 \| \| 6 \| not applicable \| 9/16/2015 15:53:35 \| \| 7 \| Yes, I have contributed to the writing of master and PhD project descriptions with focus on biodiversity informatics to be offered at the University of Oslo. So far we got one master student, but our proposed phd project was heavily restructured to include substantially less focus on (biodiversity) informatics. \| 9/16/2015 18:26:00 \| \| 8 \| It is of our interest \| 9/16/2015 19:14:40 \| \| 9 \| Not at the moment \| 9/17/2015 4:08:37 \| \| 10 \| Same course content was used during some mentoring projects. \| 9/17/2015 11:21:05 \| \| 11 \| yes but it is just a project at the moment \| 9/18/2015 16:31:38 \| \| 12 \| We would like to expand our training programme to become a regular programme for practitioners in the region but not at academic level. The way we are designing our programme is such that the programme will help build capacity of practitioners in the region on specific topics that are deemed relevant to our mission and objective \| 9/22/2015 12:03:20 \| \| 13 \| No \| 9/22/2015 13:34:08 \| \| 14 \| I am actually elaborating the curriculum and will very soon submit it to my University for acceptance \| 9/22/2015 16:07:15 \| \| 15 \| We are looking at an extended curricula (Hons) for Biodiversity Information Management and working with additional Universities to roll-out such a course. SANBI is developing a 5 year strategic plan and will engage with government for further investment. This plan includes bringing honours, Masters and PhD students on board as well as informatics expertise, at the regional and international level. \| 9/26/2015 17:48:23 \| \| 16 \| No. \| 9/30/2015 3:03:03 \| \| 17 \| no \| 10/2/2015 16:37:02 \| |
| --- | --- | --- | --- | --- | --- | --- | --- | --- | --- | --- | --- | --- | --- | --- | --- | --- | --- | --- | --- | --- | --- | --- | --- | --- | --- | --- | --- | --- | --- | --- | --- | --- | --- | --- | --- | --- | --- | --- | --- | --- | --- | --- | --- | --- | --- | --- | --- | --- | --- | --- | --- | --- | --- | --- |

# ELEARNING

**Q13. Have you been engaged in developing or implementing any e-learning modules? If yes, please provide more detail on the nature of these modules and the scope of the content**

Answered: 16 Skipped: 15

| \| \| Number \| Answer. \| Timestamp \| \| --- \| --- \| --- \| \| 1 \| No \| 9/15/2015 13:39:58 \| \| 2 \| No but I would like to participate in his kind of work \| 9/16/2015 7:07:38 \| \| 3 \| <http://www.gbif.es/eLearningGbifes_in.php> \| 9/16/2015 8:41:33 \| \| 4 \| No \| 9/16/2015 9:52:50 \| \| 5 \| Well, not e-learning in the sense that the course given is fully automated but any teaching is performed in terms of pre- set "lectures" and exercises provided ex-campus plus handled over the Internet. \| 9/16/2015 15:33:18 \| \| 6 \| Yes, but at another institution (museum). There I used Adobe Captivate. \| 9/16/2015 15:53:35 \| \| 7 \| No. However, I usually make educational slides freely available online at SlideShare, http://www.slideshare.net/dagendresen \| 9/16/2015 18:26:00 \| \| 8 \| It is of our interest \| 9/16/2015 19:14:40 \| \| 9 \| Not at the moment \| 9/17/2015 4:08:37 \| \| 10 \| We are currently implementing our e-learning platform.  The content will be on data publication, data quality and data paper \| 9/18/2015 16:31:38 \| \| 11 \| no \| 9/22/2015 10:57:20 \| \| 12 \| Yes, Certification \| 9/22/2015 13:34:08 \| \| 13 \| No \| 9/22/2015 16:07:15 \| \| 14 \| no \| 9/26/2015 17:48:23 \| \| 15 \| No \| 9/30/2015 3:03:03 \| \| 16 \| no \| 10/2/2015 16:37:02 \| \| \| --- \| --- \| --- \| --- \| --- \| --- \| --- \| --- \| --- \| --- \| --- \| --- \| --- \| --- \| --- \| --- \| --- \| --- \| --- \| --- \| --- \| --- \| --- \| --- \| --- \| --- \| --- \| --- \| --- \| --- \| --- \| --- \| --- \| --- \| --- \| --- \| --- \| --- \| --- \| --- \| --- \| --- \| --- \| --- \| --- \| --- \| --- \| --- \| --- \| --- \| --- \| --- \| |
| --- | --- | --- | --- | --- | --- | --- | --- | --- | --- | --- | --- | --- | --- | --- | --- | --- | --- | --- | --- | --- | --- | --- | --- | --- | --- | --- | --- | --- | --- | --- | --- | --- | --- | --- | --- | --- | --- | --- | --- | --- | --- | --- | --- | --- | --- | --- | --- | --- | --- | --- | --- | --- |

**Q14. Have you been involved in developing any online courses or Massive Open Online Courses (MOOCs)?**

Answered: 21 Skipped: 10


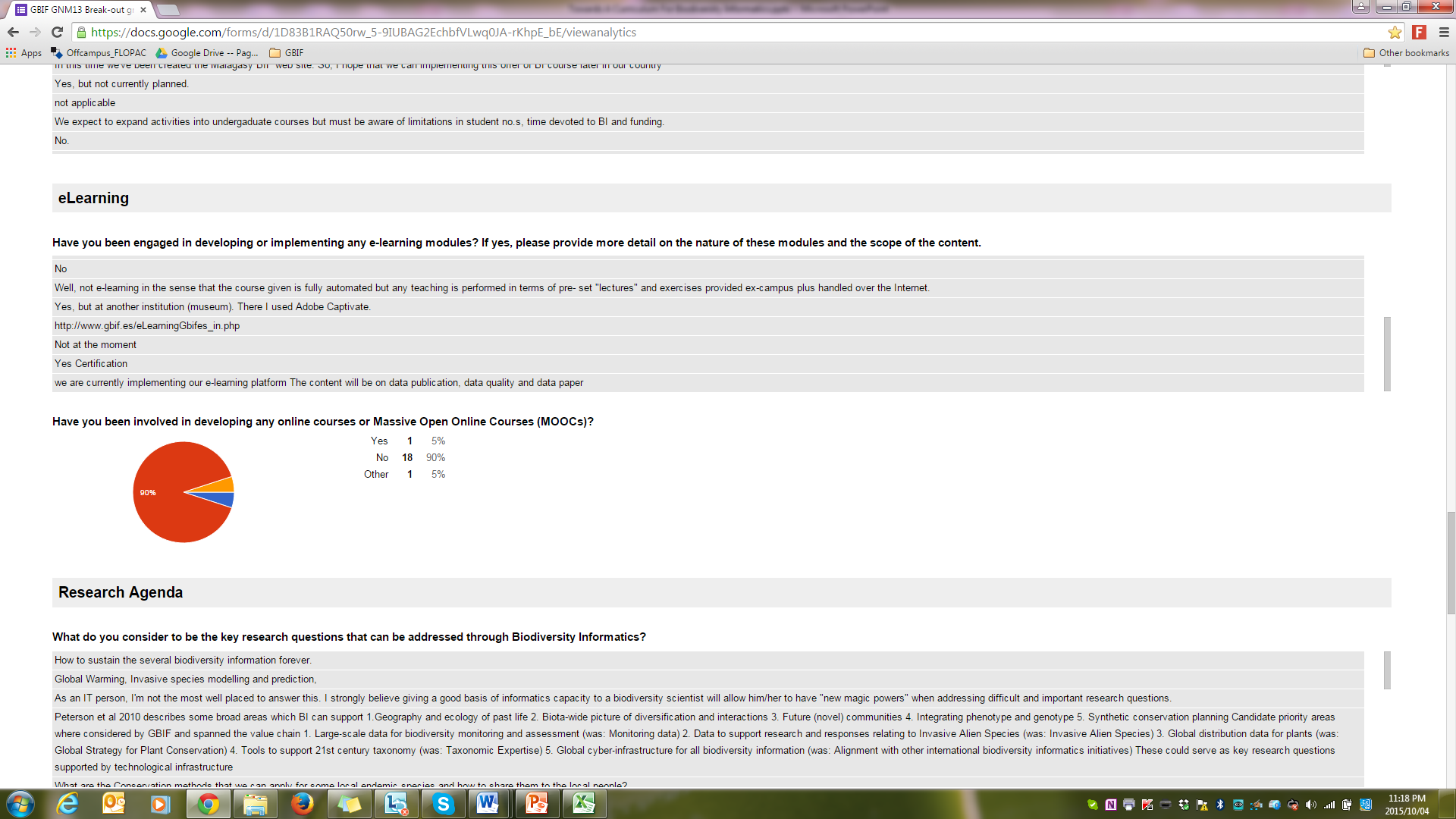


| **Answer Choices** | **Responses** | **Percentage** |
| --- | --- | --- |
| Yes | **1** | 5% |
| No | **18** | 90% |
| Other | **1** | 5% |

# RESEARCH AGENDA

**Q15. What do you consider to be the key research questions that can be addressed through Biodiversity Informatics?**

Answered: 16 Skipped: 14

| \| **Number** \| **Answer** \| **Timestamp** \| \| \| --- \| --- \| --- \| --- \| \| 1 \| Global Warming, Invasive species modelling and prediction, \| 9/15/2015 3:39:58 \| \| 2 \| I think this a wrong question. BI is a tool, a method an approach, applications are multiple... what would be your answer if instead BI you say molecular techniques? BI is allowing us --for the first time in history-- "to know what we know about biodiversity on earth"; and with that you can do a lot; from patterns and trends, to planning butterfly watching excursions... What is the big question that Google can answer? \| 9/16/2015 8:41:33 \| \| 3 \| What are the Conservation methods that we can apply for some local endemic species and how to share them to the local people? \| 9/16/2015 9:52:50 \| \| 4 \| Temporal and spatial distribution and taxonomic and phylogenetics analysis. Database structuring and data presentation. \| 9/16/2015 15:33:18 \| \| 5 \| Online identification of material, either in collections or from (other) Research projects \| 9/16/2015 15:53:35 \| \| 6 \| Biodiversity informatics contributes or even sometimes underpin a very wide section of biology related research questions!  Examples (far from exhaustive), Species Distribution Modeling, Conservation strategies, Red list, threatened species assessments, Black list, invasive species assessments, Conservation strategy for crop wild relatives, Citizen science, e.g. on involving and motivating the public, Predictive ecogeographic analysis of useful traits, Predictive analysis of genetic diversity, Barcode of life, characteristic sequences for identification of species, Characteristic sequences for identification on infraspecific level, Predictive effects of climate change on species distribution, adaptation, etc \| 9/16/2015 18:26:00 \| \| 7 \| How to facilitate data gathering on field?, How to facilitate data documenting on DarwinCore and Plinian Core?, How to publish data through IPT?, How to build and publish data papers?, How to make available data and analysis for decision making?, How to make biodiversity numbers useful for child education?, What can be get from data analysis? \| 9/16/2015 19:14:40 \| \| 8 \| Digitizing specimen records and using standardized Biodiversity data. \| 9/17/2015 4:08:37 \| \| 9 \| Climate Change and other Biodiversity threads modelling. \| 9/17/2015 11:21:05 \| \| 10 \| As an IT person, I'm not the most well placed to answer this. I strongly believe giving a good basis of informatics capacity to a biodiversity scientist will allow him/her to have "new magic powers" when addressing difficult and important research questions. \| 9/17/2015 14:04:23 \| \| 11 \| Species niche modelling responses to predict climate change, invasive species spread. \| 9/22/2015 10:57:20 \| \| 12 \| Invasive species and climate change is one of the pressing threats in our region that can be addressed through biodiversity informatics \| 9/22/2015 12:03:20 \| \| 13 \| Factors impacting species distribution in the context of global change -Ecological Niche Modelling of targeted species in food security, forest conservation, Identification of appropriate strategy for biodiversity conservation \| 9/22/2015 16:07:15 \| \| 14 \| Peterson *et al* 2010 describes some broad areas which BI can support 1. Geography and ecology of past life 2. Biota-wide picture of diversification and interactions 3. Future (novel) communities 4. Integrating phenotype and genotype 5. Synthetic conservation planning. Candidate priority areas where considered by GBIF and spanned the value chain. 1. Large‐scale data for biodiversity monitoring and assessment (was: Monitoring data) 2. Data to support research and responses relating to Invasive Alien Species (was: Invasive Alien Species) 3. Global distribution data for plants (was: Global Strategy for Plant Conservation) 4. Tools to support 21st century taxonomy (was: Taxonomic Expertise) 5. Global cyber‐infrastructure for all biodiversity information (was: Alignment with other international biodiversity informatics initiatives). These could serve as key research questions supported by technological infrastructure \| 9/26/2015 17:48:23 \| \| 15 \| As a discipline, biodiversity informatics is a methodological science. So, it should discover how to make the "big data" resource more useful to both scientist and decision makers, with current and new emerging technologies. From content point of view, this could mean development of standards and ontology models (including taxonomies), methods for annotating data with these and finally creating a mechanism for linking this additional value back to the originator of the data. One great possibility with informatics is to explore the data driven (no hypotheses) science to find out trends that are not visible in smaller data. \| 9/29/2015 16:03:09 \| \| 16 \| Help identify the status biodiversity and minimize threats to biodiversity by efficiently integrating data for analysis supporting decision and act. \| 9/30/2015 3:03:03 \| \| 17 \| How to sustain the several biodiversity information forever. \| 10/2/2015 16:37:02 \| |
| --- | --- | --- | --- | --- | --- | --- | --- | --- | --- | --- | --- | --- | --- | --- | --- | --- | --- | --- | --- | --- | --- | --- | --- | --- | --- | --- | --- | --- | --- | --- | --- | --- | --- | --- | --- | --- | --- | --- | --- | --- | --- | --- | --- | --- | --- | --- | --- | --- | --- | --- | --- | --- | --- | --- | --- |

**Q16. According to you, what are the global priorities for a research agenda in Biodiversity Informatics?**

Answered: 16 Skipped: 15

| **Number** | **Answer** | **Timestamp** | |
| --- | --- | --- | --- |
| 1 | Biodiversity Informatics should link genomics, phylogenetics, taxonomy, distributional biology, ecology, interactions, and conservation status (see also: DOI:10.1080/14772001003739369 | | 9/15/2015 13:39:58 |
| 2 | Data analytics and cleaning | | 9/16/2015 7:07:38 |
| 3 | a) Developing methods by which we can "calculate" to what taxon an individual/specimen belongs instead of having humans (experts, hopefully) "identifying" it. b) Tackling the ontological component of biodiversity informatics for real | | 9/16/2015 8:41:33 |
| 4 | How to share the documentations to the local people? | | 9/16/2015 9:52:50 |
| 5 | See above. To assure young people are made aware of their own responsibility to handle (store and present) data correctly, and to enable analysis. | | 9/16/2015 15:33:18 |
| 6 | online identification and building up databases that overcome the taxonomic impediment (meaning: speeding up the recognition and description of (new) species) | | 9/16/2015 15:53:35 |
| 7 | 1. My top priority for biodiversity informatics must be to support the implementation of persistent identifiers for species occurrences and biological collection specimens and materials in botanical gardens and genebanks. b) A second priority must be the development of data type models and controlled vocabularies for standardized terminology. | | 9/16/2015 18:26:00 |
| 8 | 1. Data publishing 2. Data visualization for decision making and results for global targets (AICHI, CBD) | | 9/16/2015 19:14:40 |
| 9 | Biodiversity data will be made available online | | 9/17/2015 4:08:37 |
| 10 | same | | 9/17/2015 11:21:05 |
| 11 | 1. Factors impacting species distribution in the context of global change 2. Ecological Niche Modeling of targeted species in food security, 3. Forest conservation... 4. Identification of appropriate strategy for biodiversity conservation | | 9/22/2015 16:07:15 |
| 12 | Documenting the location, status and trend of imperilled species and ecosystems. Developing ways to document the effectiveness of conservation actions. Developing more effective ways to provide information to guide conservation actions. | | 9/23/2015 23:32:33 |
| 13 | A number of priorities have been identified through the GBIF-Africa work for the region. Including: Invasive Aliens, marine biodiversity, native and endemic species, medicinal plants, Agrobiodiversity, Forest species. Many of these are relevant at the global level. | | 9/26/2015 17:48:23 |
| 14 | Developing the standards and vocabularies/ontologies to better describe the fitness-for-use of the data. | | 9/29/2015 16:03:09 |
| 15 | Make all kinds of data relevant to biodiversity and ecological conservation open to public and provide tools/platform for integrated analysis to evaluate and predict the trend of biodiversity in countries, regions, and the globe. | | 9/30/2015 3:03:03 |
| 16 | Filling the data gap especially in megadiverse regions. | | 10/2/2015 16:37:02 |

# POTENTIAL CONTRIBUTIONS AND OPPORTUNITIES TO GLOBALLY-OFFERED BIODIVERSITY INFORMATICS TRAINING CURRICULUM

**Q17. Would you be willing to mentor or train students or work-based professionals as part of a globally-offered biodiversity informatics programme?**

Answered: 20 Skipped: 11


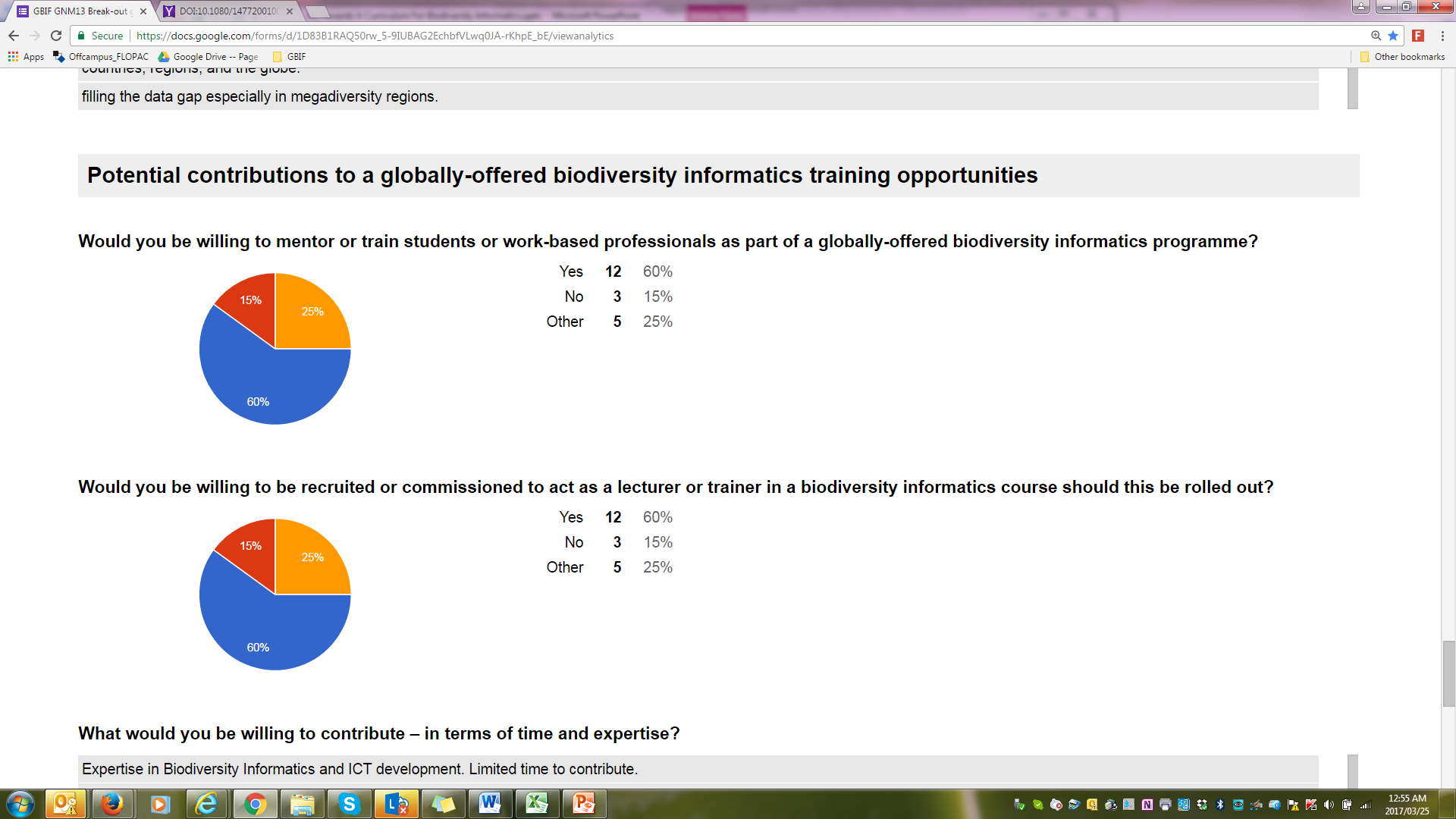


|  |  |  |  |
| --- | --- | --- | --- |
| **Answer Choices** | | **Responses** | **Percentage** |
| Yes | | 12 | 60% |
| No | | 3 | 15% |
| Other | | 5 | 25% |

**Q18. Would you be willing to be recruited or commissioned to act as a lecturer or trainer in a biodiversity informatics course should this be rolled out?**

Answered: 21 Skipped: 10


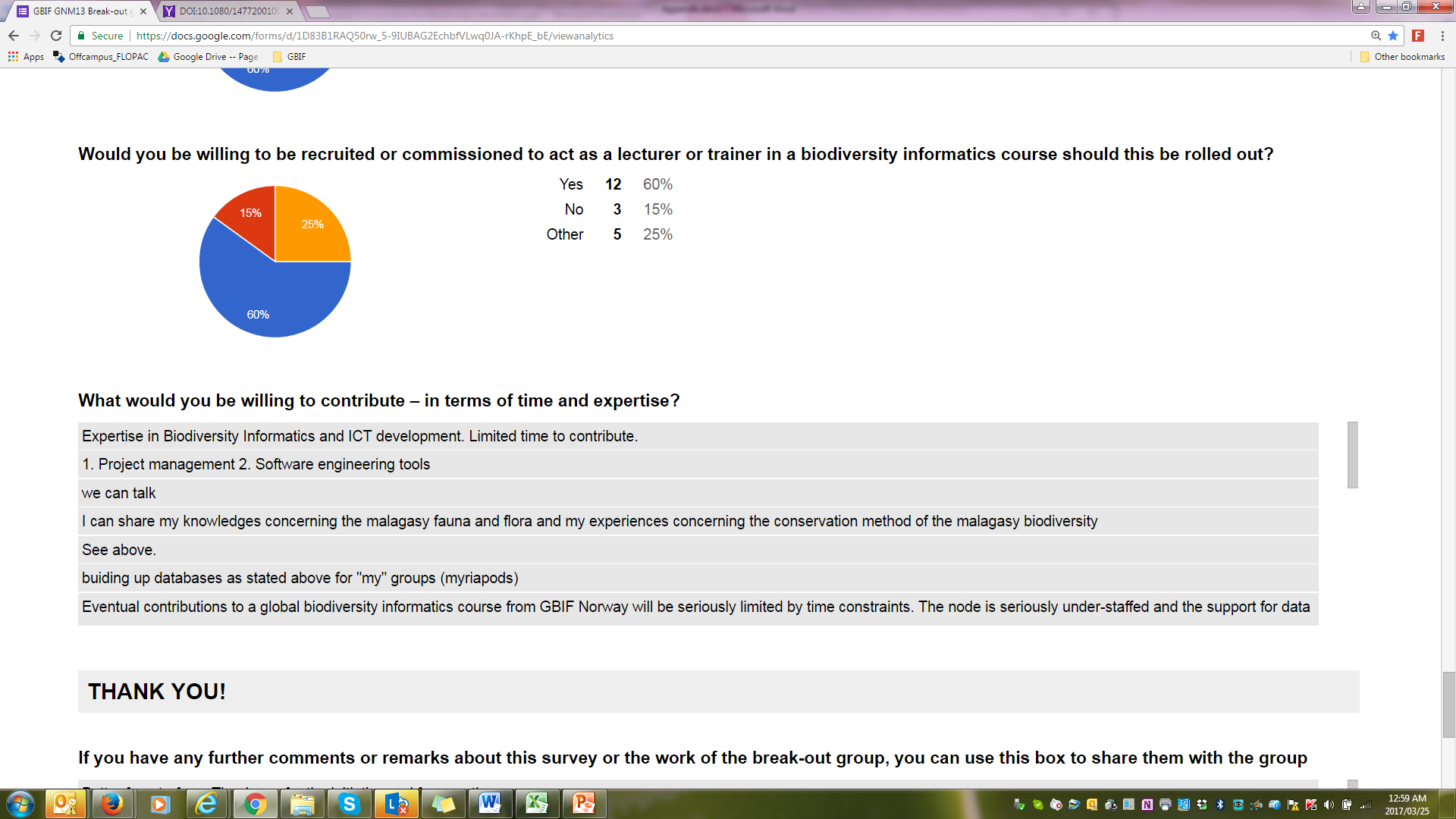


| **Answer Choices** | **Responses** | **Percentage** |
| --- | --- | --- |
| Yes | **12** | 60% |
| No | **3** | 15% |
| Other | **5** | 25% |

**Q19. What would you be willing to contribute – in terms of time and expertise?**

Answered: 19 Skipped: 12

| \| **Number** \| **Answer** \| **Timestamp** \| \| --- \| --- \| --- \| \| 1 \| Expertise in Biodiversity Informatics and ICT development. Limited time to contribute. \| 9/15/2015 13:39:58 \| \| 2 \| 1. Project management 2. Software engineering tools \| 9/16/2015 7:07:38 \| \| 3 \| we can talk \| 9/16/2015 8:41:33 \| \| 4 \| I can share my knowledges concerning the Malagasy fauna and flora and my experiences concerning the conservation method of the Malagasy biodiversity \| 9/16/2015 9:52:50 \| \| 5 \| See above. \| 9/16/2015 15:33:18 \| \| 6 \| building up databases as stated above for "my" groups (*Myriapods*) \| 9/16/2015 15:53:35 \| \| 7 \| Eventual contributions to a global biodiversity informatics course from GBIF Norway will be seriously limited by time constraints. The node is seriously under-staffed and the support for data publication remain our top priority. We want to participate but must also be very careful not to offer more time resources than we can keep. Expertise include: *Data analysis, including statistical methods; *Geographic information systems, and spatial data analysis; * Genetic resources, including predictive eco-geographic analysis; * Knowledge organization systems and principles \| 9/16/2015 18:26:00 \| \| 8 \| Data portals building; Publishing model establishment; Information architecture; Biodiversity information standards \| 9/16/2015 19:14:40 \| \| 9 \| Managing Biodiversity data \| 9/17/2015 4:08:37 \| \| 10 \| Time: 5% of my time is conceivable. Expertise: database, GIS, DwC, data cleaning \| 9/17/2015 11:21:05 \| \| 11 \| Really depends of which requests are coming and how. But the will to contribute is definitely there! \| 9/17/2015 14:04:23 \| \| 12 \| Our node is interested to contribute but difficult to estimate the time. \| 9/18/2015 16:31:38 \| \| 13 \| Biodiversity database management, online tools, citizen science \| 9/22/2015 10:57:20 \| \| 14 \| GIS; Data capture; Data cleaning and publishing \| 9/22/2015 16:07:15 \| \| 15 \| We are still developing our training program. I think we will be in a better position to provide time and expertise in two to three years. \| 9/23/2015 23:32:33 \| \| 16 \| I am flexible \| 9/26/2015 17:48:23 \| \| 17 \| I could offer my network to find out specific expertise... \| 9/29/2015 16:03:09 \| \| 18 \| Help organize regional training workshop of BI and act as a trainer. \| 9/30/2015 3:03:03 \| \| 19 \| Making many case studies including data publishing, data use for Japan (my country) and Asia regions because of the backward situation among the world \| 10/2/2015 16:37:02 \| |
| --- | --- | --- | --- | --- | --- | --- | --- | --- | --- | --- | --- | --- | --- | --- | --- | --- | --- | --- | --- | --- | --- | --- | --- | --- | --- | --- | --- | --- | --- | --- | --- | --- | --- | --- | --- | --- | --- | --- | --- | --- | --- | --- | --- | --- | --- | --- | --- | --- | --- | --- | --- | --- | --- | --- | --- | --- | --- | --- | --- | --- |

If you have any further comments or remarks about this survey or the work of the break-out group, you can use this box to share them with the group

| 1. Better face-to-face. Thank you for the initiative and for your time |
| --- |
| 1. How can we get some documentation concerning this thema: Biodiversity Informatics? |
| 1. NB! The nodes are very pressed on time. A realistic first step for development of a biodiversity informatics curriculum from within the node community might perhaps be to find funding to support the human resources and time needed. An alternative is to flag the need for biodiversity informatics training and influence the universities or university networks to give this higher priority. |
| 1. Thanks for this effort, I hope this session would be very productive and I think this kind of previous inputs can speed up the work around this topic. Also, I'm currently doing a list of courses related to data management https://goo.gl/Iiu3gs that can help to structure or complement any proposal. |
| 1. Due to lack of direct funding, the German GBIF Node system is presently carried by institutional commitments of the node institutions. There are numerous educational activities carried out by these institutions, e.g. software training for the Diversity Workbench developed at the mycological node and the EDIT Platform for Cybertaxonomy at the botanical node, which are in use also in other nodes. The GBIF node institutions also support several citizen-science as well as data mobilisation and data repository initiatives, and the botanical node is directly linked to a university. |
| 1. I recommend the work that has already been put in developing the biodiversity informatics curriculum and the materials are really a good help for stakeholders involved in capacity building activities around this topic. The effort to translate these materials is really commendable and we would like to help if a framework is devised to do so. I checked the DotSub website but I couldn't see how one can contribute at least in the translation of the videos' subtitles/captions. |
| 1. Thank you |
| 1. It's very important to evolve this group and to support each of the participants in their activities like organization of activities, fundraising, capacity building/enhancement, knowledge sharing, exchanges of trainers, etc. |
